# Indigenous gut microbiota constitutively drive release of ciliary neurotrophic factor from mucosal enteric glia to maintain the homeostasis of enteric neural circuits

**DOI:** 10.3389/fimmu.2024.1372670

**Published:** 2024-11-13

**Authors:** Ryo Kato, Takeshi Yamamoto, Hanako Ogata, Kana Miyata, Shusaku Hayashi, Michael D. Gershon, Makoto Kadowaki

**Affiliations:** ^1^ Division of Gastrointestinal Pathophysiology, University of Toyama, Toyama, Japan; ^2^ Departments of Pathology and Cell Biology, Columbia University Vagelos College of Physicians and Surgeons, New York, NY, United States

**Keywords:** enteric glia, enteric nervous system, ciliary neurotrophic factor (CNTF), gut microbiota, TLR4 (toll-like receptor 4), antibiotics, LPS (lipopolysaccharide), mucosa

## Abstract

It has recently become clear that the gut microbiota influence intestinal motility, intestinal barrier function, and mucosal immune function; therefore, the gut microbiota are deeply involved in the maintenance of intestinal homeostasis. The effects of the gut microbiota on the enteric nervous system (ENS) in the adult intestine, however, remain poorly understood. In the current study, we investigated the effects of the gut microbiota on the ENS. Male C57BL/6 SPF mice at 12 weeks of age were given a cocktail of four antibiotics (ABX) orally to induce dysbiosis (ABX mice). As early as six hours after ABX administration, the weight of the cecum of ABX mice increased to be significantly greater than that of vehicle-treated animals; moreover, ABX-induced dysbiosis reduced the density of enteric nerve fibers (marked by tubulin-β3 immunoreactivity) in the lamina propria of the proximal colon to approximately 60% that of control. TAK242, a TLR4 antagonist, significantly lowered the nerve fiber density in the lamina propria of the proximal colonic mucosa to approximately 60% that of vehicle-treated SPF mice. We thus developed and tested the hypothesis that mucosal glia expressing TLR4 are activated by enteric bacteria and release neurotrophic factors that contribute to the maintenance of enteric neural circuits. Neurotrophic factors in the mucosa of the SPF mouse proximal colon were examined immunohistochemically. Ciliary neurotrophic factor (CNTF) was abundantly expressed in the lamina propria; most of the CNTF immunoreactivity was observed in mucosal glia (marked by S100β immunoreactivity). Administration of CNTF (subcutaneously, 0.3 mg/kg, 3 doses, 2 hours apart) to ABX mice significantly increased mucosal nerve fiber density in the ABX mouse proximal colon to nearly control levels. The effect of CNTF on enteric mucosal nerve fibers was examined in isolated preparations of proximal colon of ABX mice. As it did *in vivo*, exposure to CNTF *in vitro* significantly increased enteric mucosal nerve fiber density in the ABX-treated colon. In conclusion, our evidence suggests that gut microbiota constitutively activate TLR4 signaling in enteric mucosal glia, which secrete CNTF in response. The resulting bacterial-driven glial release of CNTF helps to maintain the integrity of enteric mucosal nerve fibers.

## 1 Introduction

The intestinal tract is responsible for the transport, digestion, and absorption of ingested food; moreover, the gut simultaneously plays a critical role as a frontline interface with the external world. Consequently, the bowel must prevent loss of body protein to the enteric lumen and defend against translocation of pathogenic and commensal microorganisms from the lumen to the body. The enteric nervous system (ENS) and intestinal mucosal immune system maintain intestinal functions and homeostasis of the whole body through complicated and sophisticated interactions (1, 2). Specifically, the ENS regulates motor and secretory activity of the intestinal tract from the esophagus to the anus and both activities contribute to prevention of microbial invasion. The ENS, like the central nervous system (CNS), is composed of glial cells and a variety of neurons, including the intrinsic primary afferents (IPANs) interneurons, and motor neurons, which comprise enteric microcircuits. Together with epithelial cells that serve as sensors, the ENS is able to detect and respond appropriately to stimuli, including nutrients, gut microbiota and their metabolites within the intestinal lumen as well as to inflammation (1, 2).

Enteric glia have been postulated to play diverse roles in neuronal support, neuroprotection, neurogenesis, neuroimmune interactions, and synaptic transmission. Enteric glia, thus might participate in the regulation of gastrointestinal motility, mucosal transport, and the mucosal immune system, thereby maintaining intestinal integrity (2, 3). Enteric glia had long been thought of primarily as filler, or support for neurons; thus, their complexity, diversity, and potential roles in the physiology and pathophysiology of the gut has only recently been discovered (2, 3). It is now clear that enteric glia are located, not only within the enteric plexuses, but also within the musculature of the bowel and within the lamina propria (1–3). The complexity of glial function is still incompletely understood. Glial fibrillary acidic protein (GFAP), the calcium-binding protein, S100β, and the transcriptional activator Sox10 are used as markers for glial cells; nevertheless, no specific marker has yet been identified that distinguishes each enteric glial subtype (4). Single-cell transcriptional profiling data show that glial diversity differs between regions of the digestive tract (5, 6); however, little is known about enteric glial heterogeneity and how enteric glial specializations contribute to specific aspects of enteric physiology. Mucosal glia have not yet been characterized at the transcriptional level; therefore, the nature of the molecular diversity of mucosal glia and the functions and physiological and pathophysiological roles of mucosal glia are incompletely understood (2).

Enteric nerve fibers and elements of the enteric mucosal immune system are located just below the layer of intestinal epithelial cells, which are constantly exposed to gut microbiota; moreover, it has become clear that gut microbiota affect intestinal sensory and motor function (2). Gut microbiota also affect intestinal barrier and mucosal immune functions and they also are involved in the development and homeostasis of the bowel (7). Lineage tracing has revealed that mucosal glia are derived from progenitors that migrate to the mucosa from submucosal and myenteric ganglia in a process that is dependent on signals provided by microbiota in the enteric lumen (8).

Toll-like receptors (TLRs) are important mediators of the effects of the gut microbiota on their hosts. TLRs in the intestinal tract are expressed by dendritic cells and other immune cells of the immune system (9), enteric neurons, enteric glia, intestinal epithelial cells, and smooth muscle cells (10). There are multiple TLR subtypes; each subtype recognizes different pathogen-associated molecular patterns (9). One of the major TLRs, TLR4, recognizes lipopolysaccharide (LPS); moreover, enteric neurons (2, 10, 11) and enteric glia (2, 10, 12) have each been reported to express TLR4. Morphological abnormalities in the ENS and consequent impaired intestinal motilities have been observed in TLR4-deficient mice (13), germ-free mice (13, 14), and mice treated with antibiotics for 12 weeks to ablate the gut microbiota (13). TLR4 signaling, moreover, enhances both enteric neuronal survival and intestinal motor function (13); furthermore, gut microbiota regulate homeostasis of mucosal glia (8). These observations suggest that gut microbiota can influence the integrity of the ENS, including the phenotypes of enteric neurons, enteric glia, and the output of enteric neural circuitry. The observations also suggest that activation of TLR signaling by gut microbiota and their metabolites play an important role in the maintenance of enteric homeostasis.

Abnormalities in the total number and compositional balance of gut microbiota (dysbiosis) induced by unhealthy dietary habits, inappropriate antibiotic use, and pathogenic bacterial infection are thought to contribute to the development of intestinal diseases, such as inflammatory bowel disease (IBD) and irritable bowel syndrome (IBS) (15). These disorders are often accompanied by abnormal intestinal motility due to defects within the ENS. Elucidation of the effects of the gut microbiota on the ENS in the adult intestinal tract is therefore important for understanding the pathogenesis of intestinal disorders associated with dysbiosis and for devising appropriate means of medical intervention. The detailed mechanisms by which the gut microbiota affect the ENS, however, remains to be discerned.

We thus developed the hypothesis that TLR4 expression enables mucosal glia to recognize enteric bacteria and subsequently release a neurotrophic factor that maintains homeostasis in mucosal nerve fibers. Gershon and colleagues reported that ciliary neurotrophic factor (CNTF), a neurotrophic cytokine, has a stronger neurotrophic effect than neurotrophin-3 (NT-3) on cultured enteric neural crest-derived cells (16). Brun and colleagues, furthermore, reported that cultured enteric glia isolated from the longitudinal muscle myenteric plexus (LMMP) of the mouse small intestine express higher levels of mRNA encoding CNTF than glial cell-derived neurotrophic factor (GDNF), nerve growth factor (NGF), NT-3, neurotrophin-4/5 (NT-4/5), leukemia inhibitory factor (LIF), or brain-derived neurotrophic factor (BDNF); moreover, no mRNA encoding any known neurotrophic factor was detected in cultured macrophage/dendritic cells isolated from the LMMP of the mouse small intestine (17). We therefore undertook to investigate the possibility that CNTF might be the postulated glial neurotrophic factor in the mucosa of the SPF (specific pathogen-free) mouse proximal colon.

In the current investigation, we investigated the effects of gut microbiota on the ENS. To do so, we employed antibiotics to induce dysbiosis in adult mice. Results suggest that gut microbiota play an important role in the maintenance of mucosal nerve fibers and that mucosal glia, their expression of TLR4, and secretion of CNTF, all play roles in the mechanism.

## 2 Materials and methods

### 2.1 Animals

Male SPF C57BL/6 (SPF) mice (Japan SLC, Shizuoka, Japan), germ-free C57BL/6 mice (gifts from Dr. Shiho Fujisaka, University of Toyama; Japan SLC), TLR4-deficient SPF C57BL/6 mice (Oriental Bioservice, Kyoto, Japan), and SPF C57BL/6 mice (Oriental Bioservice) aged 12 weeks were used for experiments. Mice were kept in a constant temperature and humidity (summer: 23 ± 2°C, 55 ± 10% humidity; winter: 22 ± 2°C, 55 ± 10% humidity) at the animal facility of the University of Toyama. Water and solid feed were available ad libitum. All experiments were conducted in accordance with the “University of Toyama Animal Experiment Handling Regulations” (animal experiment plan approval number: A2018 INM-3).

### 2.2 Antibiotic-induced dysbiosis

**Figure 1:**
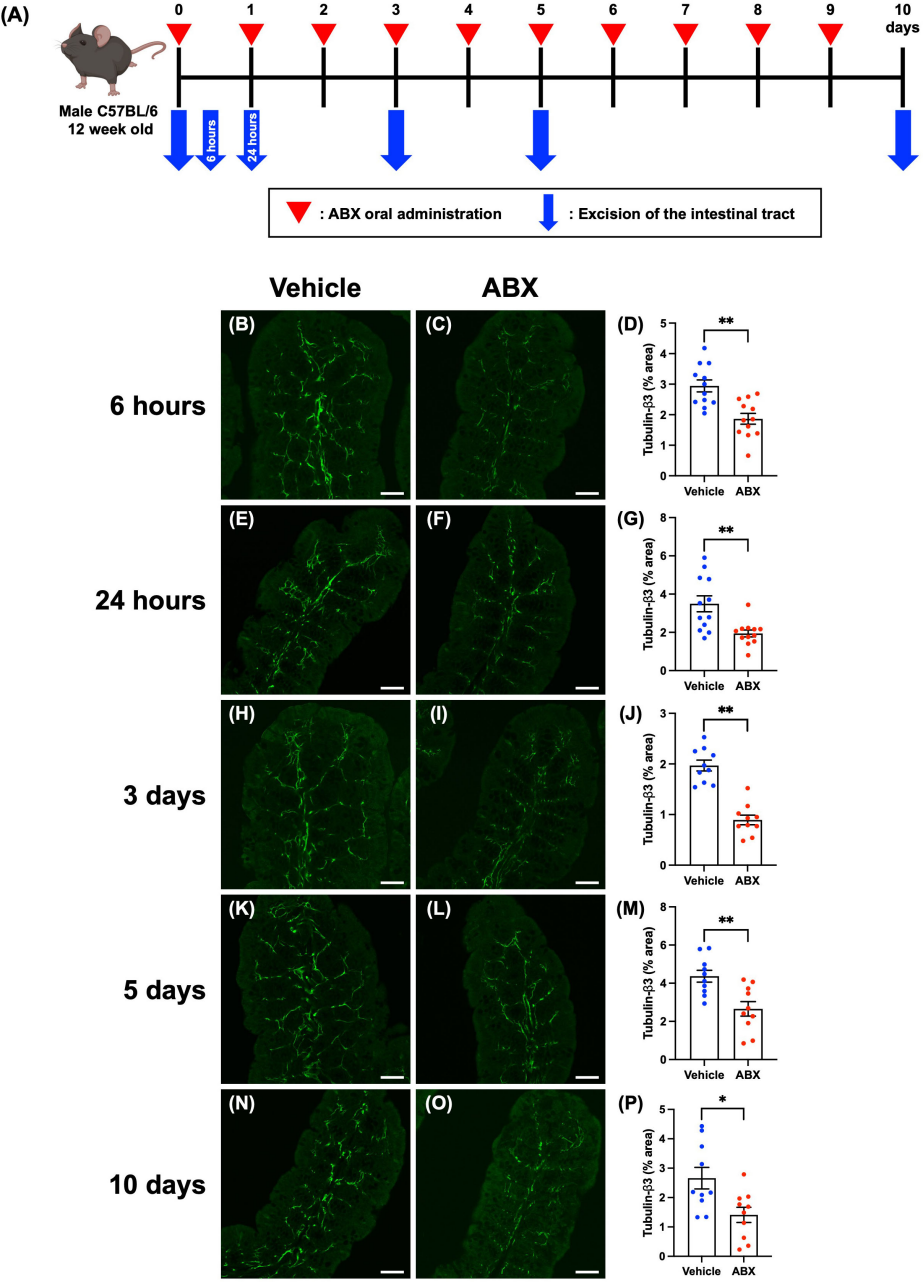
Effects of ABX-induced dysbiosis on enteric nerve fibers in the mucosal lamina propria of the proximal colon of mice. Sections of the proximal colon of vehicle-treated and ABX mice were immunostained with antibodies to tubulin-β3. Images were acquired using a Zeiss LSM780 laser-scanning confocal microscope. Workflow of ABX treatment is shown in **(A)**. This figure was created with BioRender.com. Typical images of tubulin-β3-immunoreactive nerves at each time point after the first administration of ABX are shown (**B, C**: 6 hours; **E, F**: 24 hours; **H, I**: 3 days; **K, L**: 5 days; **N, O**: 10 days). Bars show the nerve fiber density (% area) in the lamina propria (**D**: 6 hours; **G**: 24 hours; **J**: 3 days; **M**: 5 days; **P**: 10 days). The nerve fiber density was significantly lower in ABX mice than in vehicle-treated mice at all time points. Scale bar = 50 µm, *P < 0.05, **P < 0.01 vs. vehicle mice. *N* = 10–12.

Twelve-week-old SPF mice were given 200 µL of a cocktail of four antibiotics (ABX; ampicillin 1.0 g/L, metronidazole 1.0 g/L, neomycin 1.0 g/L, vancomycin 0.5 g/L) or vehicle (distilled water) orally once daily. The intestinal tracts of the mice were removed at 6 hours, 24 hours, 3 days, 5 days, or 10 days after the first ABX administration. After necropsy, the cecum was weighed, and the proximal colon was used for immunohistochemical studies. Workflow of ABX treatment is shown in **Figure 1A**.

### 2.3 Inhibition of TLR4 signaling by TAK242 administration

Twelve-week-old SPF mice were treated subcutaneously with the TLR4 inhibitor, TAK242 (3 mg/kg, ChemScene, Monmouth Junction, USA), or vehicle (0.25% DMSO). Twenty-four hours after administration of TAK242 or vehicle, their intestinal tracts were removed. After necropsy, the proximal colon was used for immunohistochemical studies.

### 2.4 Determination of the effect of ciliary neurotrophic factor on ABX-induced dysbiosis mice

ABX were administered orally to SPF mice to induce dysbiosis (ABX mice), and ciliary neurotrophic factor (CNTF; Bio Legend, San Diego, USA) was simultaneously administered subcutaneously at a dose of 0.3 mg/kg. After co-administration of ABX and CNTF (0 hours), two additional doses of CNTF were given subcutaneously at 2-hour intervals. Six hours after ABX administration, the intestinal tract was removed. After necropsy, the proximal colon was investigated immunohistochemically. Workflow of ABX and CNTF treatment is shown in figure of CNTF treatment.

In order to study the effects of CNTF on isolated preparations of colon, ABX was given orally to an additional group of mice to induce dysbiosis and the proximal colon was removed 6 hours later. The excised proximal colon was immersed in Tyrode’s solution (136.0 mM NaCl, 10.0 mM glucose, 5.4 mM KCl, 5.0 mM HEPES, 1.8 mM CaCl2, 1.0 mM MgCl2, 0.33 mM NaH2PO4; pH 7.4; 37°C), while oxygen was supplied. CNTF (100 ng/ml) was then applied to the excised proximal colon for 6 hours, and then immunohistochemical studies were carried out.

### 2.5 Immunostaining of frozen sections of the mouse intestine

Intestines dissected from C57BL/6 mice were fixed with 4% formaldehyde (from paraformaldehyde) in 0.1 M phosphate buffer, pH 7.4 for 24 hours, washed in 0.01 M PBS (pH 7.4) and stored in 30% sucrose/0.01 M PBS at 4°C as previously described (18, 19). The fixed intestines were then embedded in Tissue-Tek O.C.T. compound (Sakura Finetek Japan, Tokyo, Japan), and frozen sections of 30 μm thickness were cut in a cryostat-microtome (CM1900 UV; Leica, Nussloch, Germany). Sections were washed with 0.01 M PBS (10 min, 3 times), permeabilized with 0.3% Triton X-100/0.01 M PBS for 18 h and blocked with normal donkey serum for 30 min. The sections were washed with 0.01 M PBS (10 min, 3 times) and exposed to primary antibodies at 4°C for 18 hours (**Table 1**), followed by secondary antibodies (Jackson ImmunoResearch, West Grove, USA; Abcam, Cambridge, UK) for 2 hours at room temperature (**Table 2**). After immunostaining, the sections were mounted and sealed on glass slides using Vectashield Mounting Medium with DAPI (Vector Laboratories, Burlingame, USA). The preparations were analyzed using a Zeiss LSM700 or LSM780 confocal laser-scanning microscope (Carl Zeiss, Oberkochen, Germany).

**Table 1:** Primary antibodies used for immunohistochemistry.

Antigen	Host species	Dilution	Sources
CNTF	Goat	1:200	AF-557-NA (R&D Systems, Minneapolis, USA)
TLR4	Goat	1:100	sc-293072 (Santa Cruz, Dallas, USA)
Synaptophysin1	Guinea pig	1:500	101 004 (Synaptic Systems, Goettingen, Germany)
S100β	Rabbit	1:1,000	ab52642 (Abcam, Cambridge, UK)
Tubulin-β3	Rabbit	1:10,000	PRB-435P (Covance, Princeton, USA)
PGP9.5	Rabbit	1:500	Ab108986 (Abcam, Cambridge, UK)
CD11c	Hamster	1:100	550283 (BD Biosciences, Franklin Lakes, USA)
F4/80	Rat	1:500	MCA497GA (Bio-Rad, Hercules, USA)

**Table 2:** Secondary antibodies used for immunohistochemistry.

Antibody	Dilution	Sources
Alexa Fluor 488-donkey anti-guinea pig IgG	1:500	706-545-148 (Jackson ImmunoResearch)
Alexa Fluor 488-donkey anti-rabbit IgG	1:400	711-545-152 (Jackson ImmunoResearch)
Alexa Fluor 647-donkey anti-goat IgG	1:400	ab150131 (Abcam)
Alexa Fluor 647-donkey anti-rabbit IgG	1:500	A31573 (Invitrogen, Waltham, USA)
Cy3-donkey anti-goat IgG	1:400	705-165-147 (Jackson ImmunoResearch)
Cy3-donkey anti-rabbit IgG	1:400	711-165-152 (Jackson ImmunoResearch)
Cy3-goat anti-hamster IgG	1:1,000	127-165-160 (Jackson ImmunoResearch)
Cy3 donkey anti-rat IgG	1:400	712-166-153 (Jackson ImmunoResearch)

### 2.6 Analysis of the area reacting with primary antibodies

Immunohistochemical images of the mucosa were acquired on frozen sections using a confocal laser-scanning confocal microscope, and the immunoreactive area in the lamina propria was analyzed using the open-source software, ImageJ (ImageJ bundled with 64-bit Java 1.8.0_172; a Java-based image processing program developed at the NIH), as illustrated in **Supplementary Figure 1**. To avoid selection bias, three preparations per mouse were analyzed.

### 2.7 Statistical analysis

Data are presented as means ± SE. Statistical analyses were carried out using commercial software (Prism 10, GraphPad Software, San Diego, USA). Unpaired t test (two-tailed) with Welch’s correction was employed to compare two groups of means and One-Way ANOVA followed by Bonferroni’s correction was used to compare three groups. *P* < 0.05 was considered to indicate a significant difference.

## 3 Results

### 3.1 Morphological investigation of enteric nerve fibers in the mucosa of the ABX mouse proximal colon

Dysbiosis was induced to investigate the effects of gut microbiota on the ENS. SPF mice were treated once daily for 10 days with ABX. Workflow of ABX treatment is shown in **Figure 1A**. The cecal weight of the resulting ABX mice was significantly greater than that of control vehicle-treated mice at all time points beginning at 6 hours after ABX administration (**Supplementary Figure 2**; A: 6 hours: vehicle mice 0.37 ± 0.02 g, ABX mice 0.47 ± 0.02 g; B: 24 hours: vehicle mice 0.41 ± 0.02 g, ABX mice 0.60 ± 0.02 g; C: 3 days: vehicle mice 0.42 ± 0.03 g, ABX mice 0.89 ± 0.05 g; D: 5 days: vehicle mice 0.41 ± 0.02 g, ABX mice 0.75 ± 0.06 g; E: 10 days: vehicle mice 0.42 ± 0.02 g, ABX mice 0.80 ± 0.05 g; **P < 0.01; *n* = 10–14). Morphological differences between ABX and vehicle-treated mice were detected only in the cecum and intestinal inflammation due to ABX administration was not observed.

To investigate possible morphological effects of ABX administration on enteric neural circuits, enteric nerve fibers in the lamina propria of the proximal colon of ABX and vehicle-treated mice were immunostained with antibodies to the neural marker, tubulin-β3. No effect on the histological structure of the mucosa was observed at any time point after ABX administration; however, tubulin-β3-immunoreactive enteric nerve fibers were observed to be more sparse in ABX mice than in vehicle-treated control animals, beginning 6 hours after ABX administration and continuing until 10 days later (**Figure 1**). The proportion of the area of the lamina propria occupied by tubulin-β3-immunoreactive enteric nerve fibers (nerve fiber density) was significantly lower in ABX mice than in vehicle-treated mice at all time points (**Figure 1**; B, C, D: 6 hours: 2.94 ± 0.19% in vehicle mice, 1.87 ± 0.18% in ABX mice; E, F, G: 24 hours: 3.49 ± 0.41% in vehicle mice, 1.94 ± 0.18% in ABX mice; H, I, J: 3 days: 1.97 ± 0.11% in vehicle mice, 0.89 ± 0.10% in ABX mice; K, L, M: 5 days: 4.37 ± 0.31% in vehicle mice, 2.66 ± 0.38% in ABX mice; N, O, P: 10 days: 2.66 ± 0.36% in vehicle mice, 1.41 ± 0.28% in ABX mice; *P < 0.05, **P < 0.01 vs. vehicle mice; *n* = 10–12). This ABX-induced decline in nerve fiber density was nearly constant over all the time points after ABX administration; nerve fiber density was approximately 40% lower than that in vehicle-treated control mice. Furthermore, this decline in nerve fiber density at 6 hours after ABX administration was verified by immunohistochemistry using an antibody against another neural marker, PGP9.5 (**Supplementary Figures 3A–C**; 6 hours: 4.63 ± 0.43% in vehicle mice, 2.56 ± 0.35% in ABX mice; **P < 0.01 vs. vehicle mice; *n* = 6–10). We confirmed that the density of nerve fibers in the mucosa of the proximal colon of ABX-treated mice was approximately 60% of that of vehicle-treated mice, similar to that observed by immunohistochemistry using the tubulin-β3 antibody.

Three days after a single dose of ABX, however, nerve fiber density in the lamina propria did not differ between vehicle-treated normal mice (**Supplementary Figure 4A**) and ABX mice (**Supplementary Figure 4B**); therefore, recovery can occur after a single injection of ABX.

### 3.2 Morphological investigation of enteric nerve fibers in the mucosa of the germ-free mouse proximal colon

Germ-free mice, which are devoid of enteric bacteria throughout ontogeny and post-natal development were employed to investigate the morphology and enteric nerve density in animals that undergo postnatal development in the virtual absence of gut microbiota. Twelve-week-old germ-free C57BL/6 (GF) mice were compared to C57BL/6 SPF animals. GF mice were significantly slightly heavier than SPF mice of the same age (**Supplementary Figure 5A**; SPF mice: 25.2 ± 0.5 g, GF mice: 28.0 ± 0.9 g; *P < 0.05; *n* = 6). The cecum was also significantly larger and heavier (~25-fold) than in SPF mice (**Supplementary Figure 5B**; SPF mice: 0.24 ± 0.02 g, GF mice: 5.04 ± 0.48 g; **P < 0.01; *n* = 6). The length of the colon of GF mice, moreover, was greater than that of SPF mice (**Supplementary Figure 5C**; SPF mice: 7.54 ± 0.15 cm, GF mice: 8.85 ± 0.23 g; **P < 0.01; *n* = 5–6). No other morphological changes or inflammation were observed in the intestine of GF mice.

**Figure 2:**
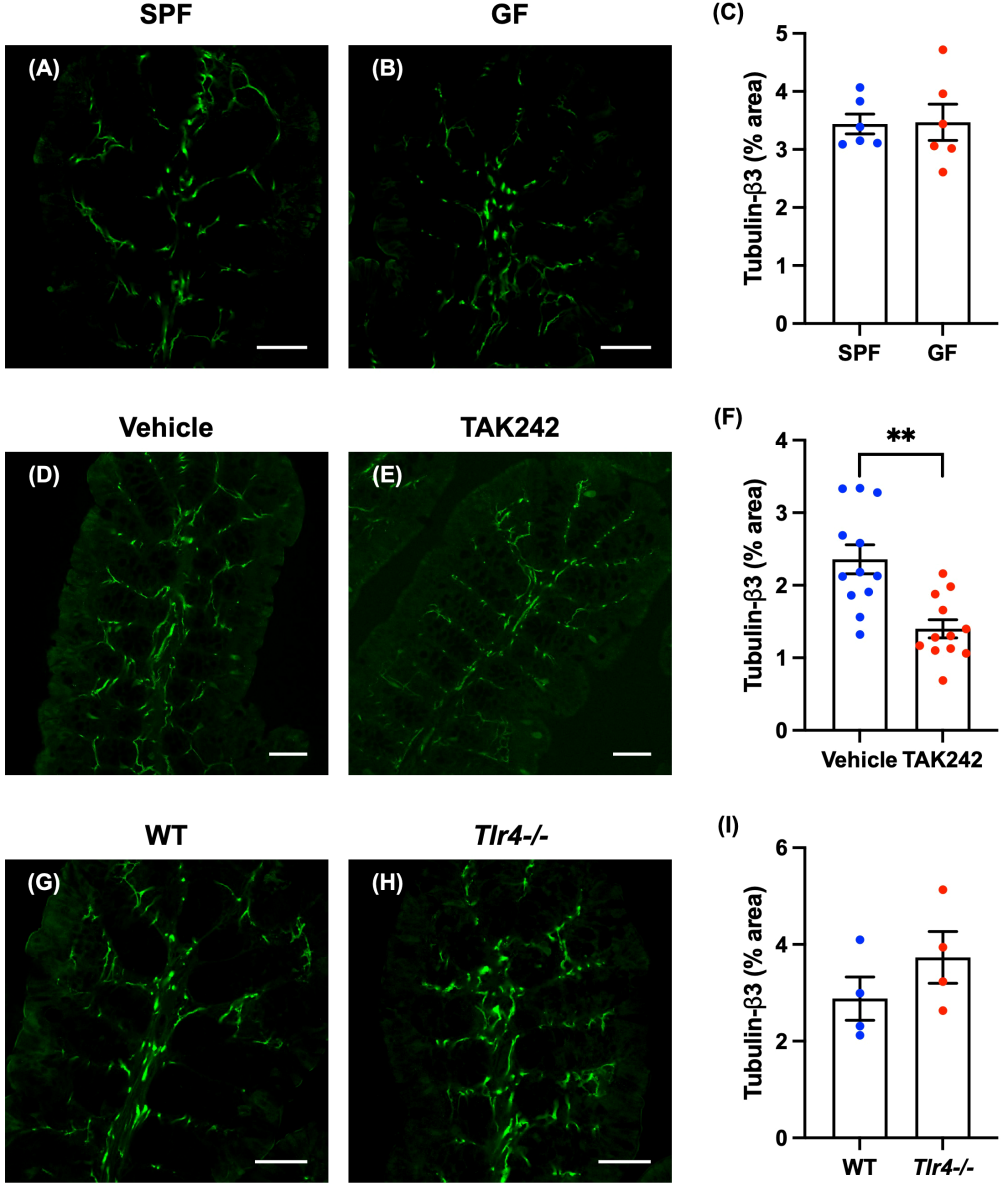
Effect of TLR4 signaling on enteric nerve fibers in the lamina propria of the mouse proximal colon. Sections of the proximal colon of SPF and GF mice were immunostained with antibodies to tubulin-β3. Images were acquired using a Zeiss LSM700 laser-scanning confocal microscope. Typical images of tubulin-β3-immunoreactive nerve fibers in the lamina propria of the proximal colon of SPF **(A)** and GF **(B)** mice are shown. Bars indicate tubulin-β3-immunoreactive nerve fiber density (% area). No significant difference in nerve fiber density **(C)** between GF and SPF mice was detected. Scale bar = 50 µm. *N* = 6. Sections of proximal colon from vehicle- and TAK242-treated mice were immunostained with antibodies to tubulin-β3. A Zeiss LSM780 laser-scanning confocal microscope was used for image acquisition. Typical images of tubulin-β3-immunoreactive nerve fibers 24 hours after administration of vehicle **(D)** or TAK242 **(E)** are shown. Bars indicate nerve fiber density (% area) in the lamina propria shown in the images **(F)**. Nerve fiber density was significantly lower in TAK242-treated mice than in control animals given vehicle **(F)**. Scale bar = 50 µm. **P < 0.01. N = 12. Sections of the proximal colon of WT and *Tlr4-/-* mice were immunostained with antibodies to tubulin-β3. Images were acquired with a Zeiss laser-scanning LSM700 confocal microscope. Typical images of tubulin-β3-immunoreactive nerve fibers in the lamina propria of the proximal colon of WT **(G)** and *Tlr4-/-* **(H)** mice are shown. Bars indicate nerve fiber density (% area) in the image **(I)**. Nerve fiber density **(I)** did not differ significantly in *Tlr4-/-* and WT mice. Scale bar = 50 µm. *N* = 4.

Enteric nerve fibers in the proximal colon of SPF and GF mice were immunostained with antibodies to tubulin-β3 to investigate nerve fiber density in the lamina propria. No differences were observed between GF mice and SPF mice in the distribution and morphology of tubulin-β3-immunoreactive nerve fibers (**Figure 2A**: SPF mice; **Figure 2B**: GF mice) or in the density of nerve fibers (**Figure 2C**; SPF mice: 3.44 ± 0.17%, GF mice: 3.47 ± 0.31%; *n* = 6).

### 3.3 Effect of TLR4 signaling on enteric nerve fibers in the mucosa of the SPF mouse proximal colon

#### 3.3.1 Morphological investigation of enteric nerve fibers in the mucosa of TLR4 inhibitor-treated mouse proximal colon

Anitha et al. showed that defective TLR4 signaling causes intestinal dysmotility (13). They also found a decrease in intestinal motility in mice treated with antibiotics, but the suppressive effect of antibiotics on intestinal motility was not observed in mice deficient in TLR4 signaling (13), suggesting that TLR4 signaling plays an important role in intestinal motility. We therefore investigated the effect of TLR4 signaling on enteric nerve fiber density. To do so, we administered TAK242, a TLR4 inhibitor (20), or vehicle (control) subcutaneously (3 mg/kg) to 12-week-old SPF mice. Twenty-four hours after administration of TAK242, the body weight of TAK242-treated mice (TAK242 mice) was not significantly different from vehicle-treated control mice; furthermore, cecal weights did not differ significantly between the two groups of mice (**Supplementary Figure 6**; vehicle mice: 0.45 ± 0.02 g, TAK242 mice: 0.44 ± 0.02 g; *n* = 13–14). No morphological changes or inflammation were observed in the intestine after TAK242 administration.

Enteric nerve fibers in the proximal colon of vehicle- and TAK242-treated mice were immunostained with antibodies to tubulin-β3 to investigate the morphology of identified nerve fibers in the lamina propria. In vehicle-treated mice, tubulin-β3-immunoreactive nerve fibers were densely distributed (**Figure 2D**). In animals treated with TAK242, however, tubulin-β3-immunoreactive nerve fibers were relatively sparse (**Figure 2E**). The density of tubulin-β3-immunoreactive nerve fibers was significantly lower in TAK242-treated mice than in vehicle-treated control animals (**Figure 2F**; vehicle mice: 2.36 ± 0.19%, TAK242 mice: 1.40 ± 0.12%; **P < 0.01; *n* = 12).

#### 3.3.2 Morphological investigation of enteric nerve fibers in the mucosa of the proximal colon of TLR4-deficient mice

We investigated enteric nerve fibers in 12-week-old TLR4-deficient C57BL/6 mice (*Tlr4-/-* mice) and SPF C57BL/6 mice (WT mice) (Oriental Bioservice, Kyoto, Japan). The weights of *Tlr4-/-* mice were significantly slightly greater than those of WT mice (**Supplementary Figure 7A**; WT mice: 25.7 ± 0.3 g, *Tlr4-/-* mice: 27.7 ± 0.2 g; **P < 0.01; *n* = 4). The cecal weights of *Tlr4-/-* mice tended to be greater than those of WT mice, but the difference was not significant (**Supplementary Figure 7B**; WT mice: 0.42 ± 0.04 g, *Tlr4-/-* mice: 0.56 ± 0.05 g; *n* = 4). Colon lengths did not differ significantly between WT mice and *Tlr4-/-* mice (**Supplementary Figure 7C**; WT mice: 8.78 ± 0.20 cm, *Tlr4-/-* mice: 9.35 ± 0.18 cm; *n* = 4). No morphological differences from WT or inflammation were observed in the intestines of *Tlr4-/-* mice.

Nerve fibers in the mucosae of *Tlr4-/-* and WT proximal colon were identified by immunostaining with antibodies to tubulin-β3 to investigate the morphology of enteric nerve fibers in the lamina propria. The distribution and morphology of tubulin-β3-immunoreactive nerve fibers were not significantly different in *Tlr4*-/- (**Figure 2H**) and WT mice (**Figure 2G**); neither was there a significant difference in nerve fiber density (**Figure 2I**; WT mice: 2.88 ± 0.45%, *Tlr4*-/- mice: 3.73 ± 0.54%; *n* = 4).

### 3.4 Morphological investigation of mucosal CNTF expression in the proximal colon of SPF mice

#### 3.4.1 TLR4-expressing mucosal glia express CNTF in the lamina propria

Since our present studies have indicated that TLR4 is deeply involved in the maintenance of homeostasis of enteric neural circuits in the mucosa, we tested the hypothesis that under normal conditions, but not under pathologically abnormal conditions as in *Tlr4-/-* mice, cells in the colonic mucosa expressing TLR4 are activated by enteric bacteria and release neurotrophic factors that contribute to the maintenance of enteric neural circuits. Therefore, we first studied on mucosal glial cells, which have recently been shown to express TLR4 and regulate intestinal homeostasis by controlling intestinal barrier function and epithelial regeneration through the release of molecular mediators including neurotrophic factors (2).

**Figure 3:**
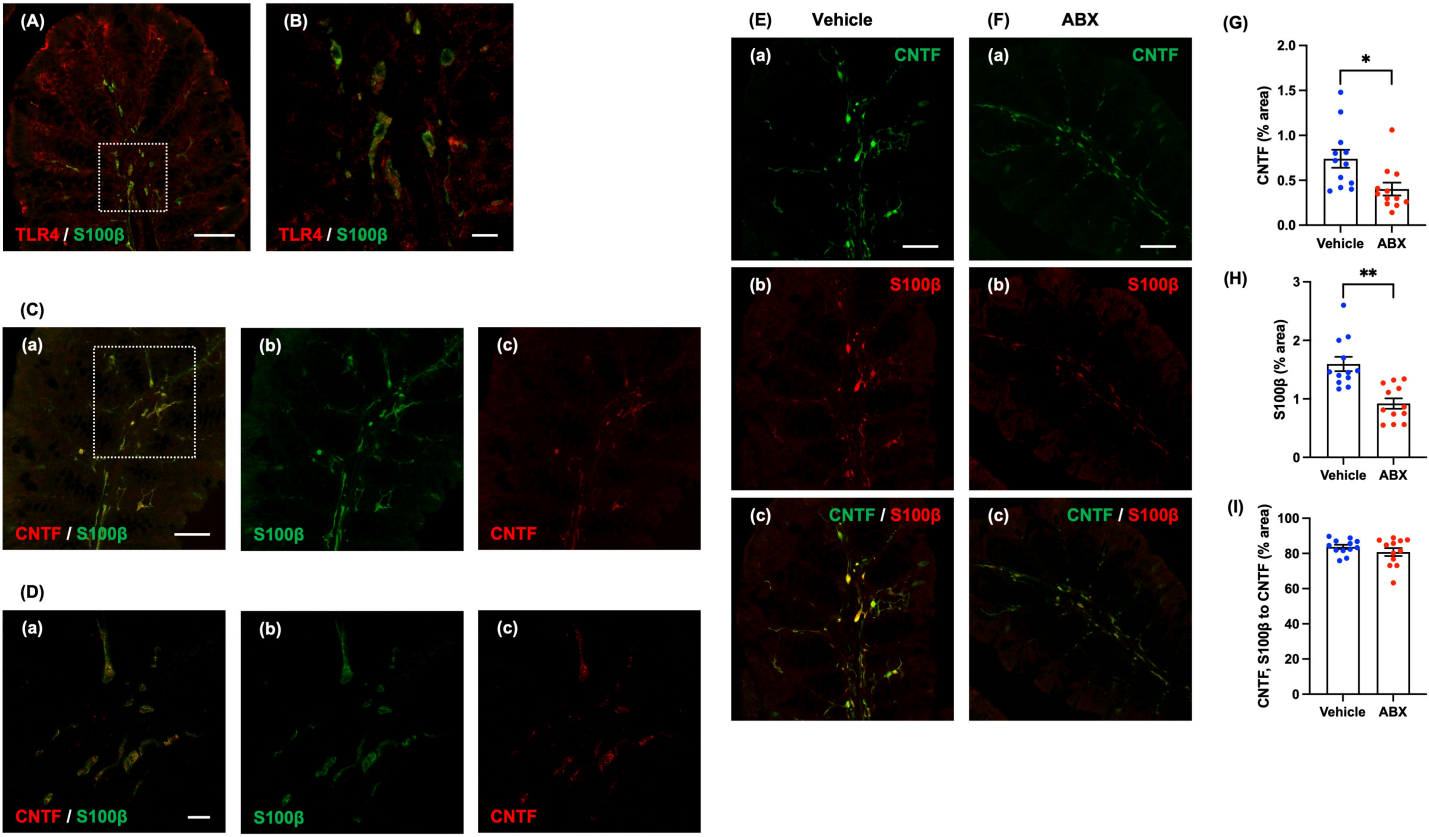
Expression of CNTF immunoreactivity in mucosal glia of the lamina propria of the mouse proximal colon. Sections of the normal mouse proximal colon were immunostained with antibodies to S100β and antibodies to TLR4. A Zeiss LSM780 laser-scanning confocal microscope was used for image acquisition. **(A)** TLR4 (red) immunoreactivity was observed in almost all S100β (green)-immunoreactive glia in the lamina propria of the proximal colon. Scale bar = 50 µm. **(B)** shows an enlarged image of the region shown in the dashed square in **(A)**. Scale bar = 10 µm. Sections of the proximal colon of SPF mice were immunostained with antibodies to CNTF and antibodies to S100β. Images were acquired using a Zeiss LSM700 laser-scanning confocal microscope. Typical images of S100β (green)-immunoreactive enteric glia (**C**b, **D**b), CNTF (red) immunoreactivity (**C**c, **D**c) in the lamina propria and merged images (**C**a, **D**a) are shown. **(D)** shows an enlarged image of the region depicted by the dashed square in **(C)**. CNTF immunoreactivity was expressed in the lamina propria of the proximal colon of SPF mice, mostly in S100β-immunoreactive enteric glia (**C**a, **D**a). Scale bar = 50 µm **(C)**, 10 µm **(D)**. Sections of the proximal colon of vehicle-treated **(E)** and ABX **(F)** mice were immunostained with antibodies for immunohistochemical analysis. Images were acquired using a Zeiss LSM700 laser-scanning confocal microscope. Typical images of CNTF (green; **E**a, **F**a) and S100β-immunoreactive glia (red; **E**b, **F**b) in the lamina propria and merged images (**E**c, **F**c) are shown. Bars indicate the proportion of the area of lamina propria that immunoreactive structures occupy in the image (% area) **(G, H)** or the proportional area that both CNTF- and S100β-immunoreactive structures occupy relative to the area of CNTF-immunoreactive structures (% area) **(I)**. The CNTF-immunoreactive **(G)** and the S100β-immunoreactive areas **(H)** were significantly lower in ABX than in vehicle-treated mice; however, the ratios of the area of CNTF- and S100β-immunoreactivities to the CNTF-immunoreactive area in the two types of mice did not differ significantly **(I)**. Scale bar = 50 µm, *P < 0.05, **P < 0.01 vs. vehicle mice. *N* = 12.

We used immunohistochemistry to investigate the location of TLR4 in the mucosa of the normal mouse proximal colon. TLR4 immunoreactivity was found to be abundant in the lamina propria; moreover, double immunostaining with antibodies against TLR4 and antibodies against S100β to identify enteric glia revealed that most mucosal glia were TLR4-immunoreactive (**Figures 3A, B**). The result is consistent with a previous report (2). We focused on CNTF as a neurotrophic factor, which has been reported to be a more potent as a neurotrophic factor than NT-3 when tested in cultured enteric neural progenitor cells (16).

Immunohistochemical staining of the proximal colon of SPF mice revealed CNTF immunoreactivity in the lamina propria (**Figure 3D** shows an enlargement of the region in the dashed square in **Figure 3C**). Double immuostaining with antibodies against S100β and antibodies against CNTF revealed that most of the CNTF immunoreactivity in the mucosa was present in S100β-immunoreactive enteric glia (**Figures 3C**a, **D**a). In contrast, no expression of CNTF immunoreactivity was observed in CD11c-immunoreactive dendritic cells, which are major immune regulators capable of sensing and responding to abnormalities and pathological changes in the intestinal lumen (**Supplementary Figure 8**).

**Figure 4:**
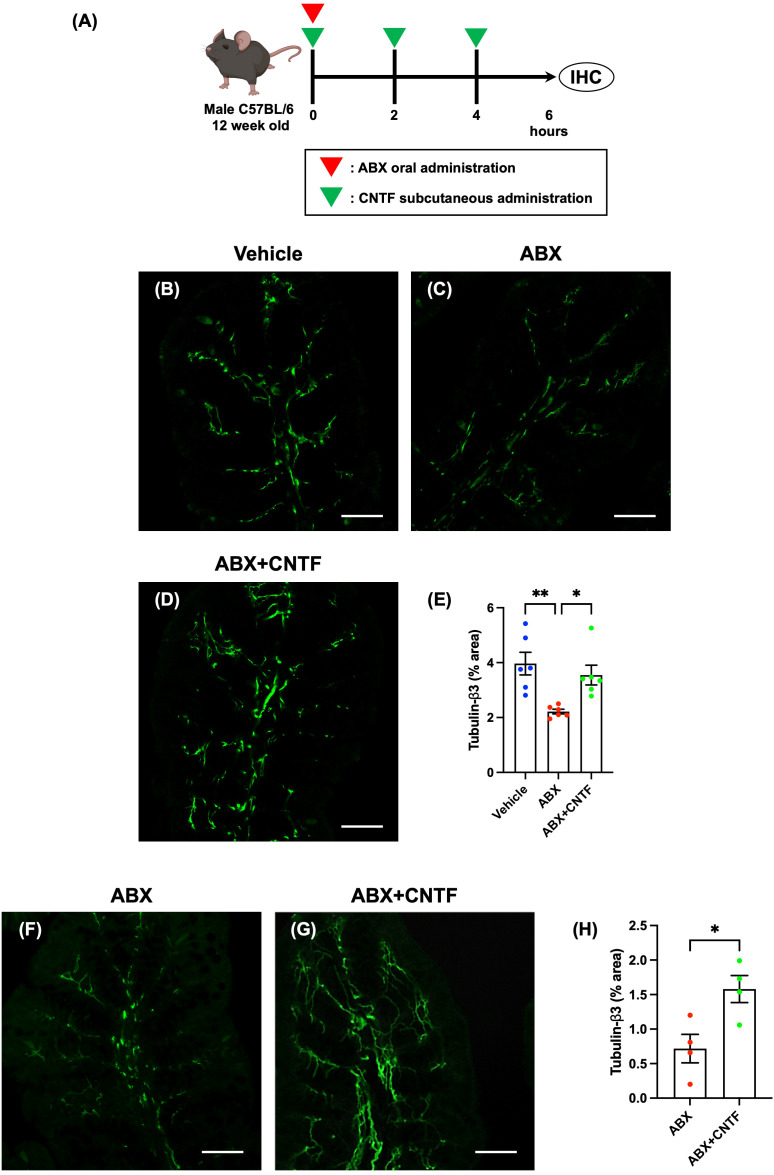
CNTF opposes the ABX-induced reduction in enteric nerve fibers in the lamina propria of the proximal colon *in vivo* and *in vitro.* SPF mice were given ABX orally to induce dysbiosis and simultaneously were treated subcutaneously with CNTF. Workflow of ABX and CNTF treatment is shown in **(A)**. This figure was created with BioRender.com. Sections of the proximal colon of vehicle-treated mice **(B)**, ABX mice **(C)** and ABX+CNTF mice **(D)** were immunostained with an antibody to tubulin-β3. Images were acquired using a Zeiss LSM700 laser-scanning-scanning confocal microscope. Typical images of tubulin-β3-immunoreactive nerve fibers 6 hours after co-administration of CNTF and ABX are shown (**B**: vehicle mice; **C**: ABX mice; **D**: ABX+CNTF mice). Bars indicate nerve fiber density (% area) in the lamina propria **(E)**. Enteric nerve fiber density was significantly lower in ABX mice than in vehicle-treated mice (**E**; **P < 0.01), but the ABX-induced decrement in nerve fiber density was virtually blocked by co-administration of CNTF (**E**; *P < 0.05 between ABX and ABX+CNTF) and the difference in nerve fiber density between ABX+CNTF and Vehicle was not significant. Scale bar = 50 µm. *N* = 6. Sections of isolated proximal colon of ABX mice **(F)** and ABX+CNTF mice **(G)** were immunostained with antibodies to tubulin-β3. Images were acquired using a Zeiss LSM700 laser-scanning confocal microscope. Typical images of tubulin-β3-immunoreactive enteric nerve fibers are shown in **(F, G)**. Bars indicate the nerve fiber density (% area) in the lamina propria **(H)**. The nerve fiber density in the lamina propria was significantly greater in preparations exposed to CNTF than to vehicle (**H**; *P < 0.05). Scale bar = 50 µm. *N* = 4.

#### 3.4.2 Anatomical proximity between of enteric glia, enteric nerve fibers and dendritic cells in the mucosa of the proximal colon in SPF mice

The proximal colon of SPF mice was doubly immunostained with antibodies to S100β and synaptophysin1. Synaptophysin1, which is often utilized as a specific marker for neuronal synaptic terminals, is the most abundant integral membrane protein of neuronal synaptic vesicles; however, synaptophysin1 is also present in a wide spectrum of neuroendocrine cells, which like neurons, have secretory vesicles (21). We found that the immunoreactivity of synaptophysin1 was coincident with that of S100β in many mucosal glial cells (**Supplementary Figures 9A, B**). This pattern of immunoreactivity is consistent both with the presence of nerve fibers containing synaptic vesicles within mesaxons of supporting glia or with presence of synaptophysin1 immunoreactivity within glial cytoplasm. We also found synaptophysin1 immunoreactivity in neuroendocrine-like cells (nonneuronal cells) of the enteric epithelial cell layer (arrows in **Supplementary Figures 9A, B**).

Dendritic cells are thought to be the primary component of the immune system that recognizes luminal information in the mucosa of the proximal colon. We therefore investigated the anatomical proximity between mucosal glia and dendritic cells in the lamina propria of the proximal colon of SPF mice. Double immunostaining with antibodies to S100β and the dendritic cell marker, CD11c, revealed that many CD11c-immunoreactive dendritic cells were closely apposed to S100β-immunoreactive mucosal glial cells (**Supplementary Figures 9C, D**).

#### 3.4.3 CNTF and mucosal glia of the proximal colon of ABX mice

We investigated the effects of oral administration of ABX (6 hours) on the immunoreactivities of CNTF and a marker of mucosal glia in the proximal colon. CNTF immunoreactivity was less abundant after treatment with ABX (**Figure 3F**a) than it was in vehicle-treated mice (**Figure 3E**a); moreover, the percent of the area of the lamina propria occupied by CNTF-immunoreactive structures was significantly lower in ABX than in vehicle-treated mice (**Figure 3G**; vehicle mice: 0.74 ± 0.10%, ABX mice: 0.40 ± 0.07%; *P < 0.05; *n* = 12). In addition, the immunoreactivity of S100β in the lamina propria of ABX mice (**Figure 3F**b) was less than that in mice treated with vehicle (**Figure 3E**b); moreover, the percent of lamina propria area occupied by S100β-immunoreactive structures was significantly lower in ABX- than in vehicle-treated mice (**Figure 3H**; vehicle mice: 1.60 ± 0.12%, ABX mice: 0.92 ± 0.90%; **P < 0.01; *n* = 12). CNTF immunoreactivity, nevertheless, was mostly expressed in S100β-immunoreactive mucosal glia in both ABX- (**Figure 3F**c) and vehicle-treated mice (**Figure 3E**c); the percent of the area of CNTF- and S100β immunoreactivities to the CNTF-immunoreactive area in the two types of mice did not differ significantly (**Figure 3I**; vehicle mice: 83.7 ± 1.2%, ABX mice: 80.8 ± 2.3%; *n* = 12).

Double immunostaining of the proximal colons of ABX and vehicle-treated mice with antibodies to CD11c and F4/80 revealed that the number of CD11c-positive-F4/80-negative dendritic cells in the lamina propria did not differ in the two types of mouse (**Supplementary Figure 10**; vehicle mice: 17.0 ± 4.6 cells/mm^2^, ABX mice: 20.8 ± 1.6% cells/mm^2^; *n* = 6). ABX treatment thus reduced the number of S100β-immunoreactive glia in the lamina propria without altering dendritic cells. The ABX-induced reduction in numbers of mucosal glial cells, which express CNTF, provides an explanation for the ability of ABX to attenuate mucosal CNTF expression.

### 3.5 Administration of CNTF opposes the ABX-induced reduction of enteric nerve fiber density in the proximal colonic mucosa

CNTF was administered to mice subcutaneously and ABX were simultaneously given orally (ABX+CNTF mice). The effect of simultaneous CNTF on the ABX-induced reduction in nerve fiber density was investigated in the lamina propria of the proximal colon. Colon length was not significantly different in ABX, ABX+CNTF, and vehicle-treated mice (*n* = 6–7). The cecum, however, was enlarged in ABX and ABX+CNTF mice and significantly heavier than that of vehicle-treated animals (**Supplementary Figure 11**; vehicle mice: 0.19 ± 0.01 g, ABX mice: 0.29 ± 0.02 g, ABX+CNTF mice: 0.33 ± 0.01 g; **P < 0.01 between vehicle mice and ABX mice, and between vehicle mice and ABX+CNTF mice; *n* = 6–7), but no effect of CNTF administration on the cecum was observed. In addition, except for the cecal enlargement in mice receiving ABX, no morphological changes or inflammation were observed in the intestines of any of the animals.

Enteric nerve fibers in the proximal colon of vehicle-treated, ABX, and ABX+CNTF mice were immunostained with antibodies to tubulin-β3 to investigate the morphology of enteric nerve fibers in the lamina propria. Enteric nerve fibers were significantly more abundant in vehicle-treated (**Figure 4B**) than in ABX mice (**Figure 4C**); moreover, the nerve fiber density in ABX mice was reduced by approximately 45% (**Figure 4E**). In contrast, in the ABX+CNTF mice (**Figure 4D**), the effect of ABX was blocked and the enteric nerve fiber density was nearly equivalent to that of vehicle-treated control animals (**Figure 4E**; vehicle mice: 3.96 ± 0.41%, ABX mice: 2.22 ± 0.08, ABX+CNTF mice: 3.55 ± 0.36; *P < 0.05 between ABX mice and ABX+CNTF mice, **P < 0.01 between ABX mice and vehicle mice; *n* = 6).

### 3.6 Effect of CNTF on nerve fibers in the mucosa of isolated preparations of proximal colon of ABX mice

The proximal colon of ABX mice was excised and incubated in an organ bath *in vitro*. The isolated colon was exposed to CNTF to determine whether the ability of CNTF to oppose the ABX-induced reduction of nerve fibers in the lamina propria was a locally mediated direct action of CNTF. *In vitro* exposure to CNTF significantly increased enteric nerve fiber density in the isolated proximal colon of ABX mice (**Figures 4F–H**; ABX: 0.71 ± 0.21, ABX+CNTF: 1.58 ± 0.20; *P < 0.05 between ABX and ABX+CNTF; *n* = 4). This observation suggests that CNTF acts directly on the colon to prevent the loss of mucosal nerve fibers due to administration of ABX.

## 4 Discussion

In the current study, we found that the density of nerve fibers, immunohistochemically identified in the lamina propria of the proximal colon with antibodies to tubulin-β3, is rapidly reduced following ABX administration; moreover, inhibition of TLR4 signaling similarly decreases the density of mucosal nerve fibers. These observations suggest that enteric bacteria, which activate TLR4, may use TLR4 signaling to contribute to the homeostasis of mucosal nerve fibers. We also demonstrated that enteric glia, identified in the lamina propria of the proximal colon with antibodies to S100β, express both TLR4 and CNTF immunoreactivities; moreover, ABX administration reduces the number of CNTF-immunoreactive mucosal glia. In contrast, exogenous CNTF co-administration with ABX opposes the ability of ABX to reduce numbers of mucosal nerve fibers and, because the effect of CNTF is manifested in isolated preparations of colon, the ability of CNTF, which promotes nerve fiber outgrowth in the lamina propria, to oppose the ABX-induced reduction of mucosal nerve fibers must be a local effect mediated directly within the colon. These observations are consistent with the hypothesis that components of the enteric microbiome, which are sensitive to ablation with ABX, activate TLR4 signaling in mucosal glia, which in turn, secrete CNTF to maintain homeostasis in nerve fibers of the enteric mucosa. This pathway may play a role in preserving the integrity of the ENS and thus be essential to optimize the physiology of the bowel.

### 4.1 Enteric neural circuits in ABX mice

In the current study, we used ABX to induce a dysbiosis in order to gain insight into the effects of enteric bacteria on the ENS. Nerve fiber density in the lamina propria of the proximal colon was decreased remarkably quickly following ABX administration and was apparent as early as 6 hours after a single dose of ABX. Nerve fiber density remained diminished through day 10 of a period of daily ABX administration. The effects of ABX-induced dysbiosis on the ENS have previously been studied, and 3 weeks of ABX treatment has been reported not to alter the number of neurons in the myenteric plexus in adult colon; however, 3 weeks of ABX treatment did decrease the density of tubulin-β3-immunoreactive nerve fibers in the myenteric plexus and lamina propria (22). The current observations and those reported previously (22) thus suggest that ABX-sensitive enteric bacteria contribute to the maintenance and/or the formation of adult enteric neural circuits. Previous investigations with an ABX-induced dysbiosis model, however, as described in a review by Kennedy and colleagues (23), have mostly investigated long-term ABX treatment of more than 3 weeks or ABX treatment of at least 3 day. For the first time, the present study shows that the enteric nerve fiber density in the colonic mucosa is reduced even 6 hours after a single administration of ABX.

It has also been reported that the total number and compositional balance of bacteria in the gut microbiota change dramatically 24 hours after ABX administration (24). In the present study, furthermore, the increase in cecal weight 6 hours after ABX administration indicates that acute dysbiosis is already induced within 6 hours of ABX administration. ABX affect the gut microbiota, therefore, promptly after their administration, and the ensuing acute dysbiosis immediately reduces the nerve fiber density in the lamina propria. ABX-induced dysbiosis thus exerts a great effect on enteric neural circuits that does not require evolution over an extended period time, but instead occurs acutely, suggesting that enteric bacteria may continuously contribute to the maintenance or the outgrowth of enteric nerve processes.

### 4.2 TLR4 signaling in the gut

It is well known that LPS, a cell wall constituent of gram-negative bacteria, affects organisms via TLR4 signaling (9). In the current study, to investigate whether activation of TLR4 signaling contributes to the effects of enteric bacteria on enteric nerve processes in the lamina propria, we administered a TLR4 inhibitor to adult mice. We found that TLR4 inhibition decreases enteric nerve fiber density in the lamina propria of the proximal colon. We then investigated the effects on the ENS of long-term loss of enteric bacteria or TLR4 signaling during ontogeny and development. To do so, we used respectively, adult germ-free and *Tlr4*-/- mice. We found that neither of these long-term defects affected enteric nerve fiber density in the lamina propria of the proximal colon.

De Vadder and colleagues reported previously that the number of neurons in the myenteric plexus of the colon and the density of tubulin-β3-immunoreactive nerve fibers in the layer of the LMMP as well as the lamina propria of the colon were not significantly different in adult germ-free and conventional mice (22). In contrast, the number of neurons in the myenteric plexus of germ-free mice has been reported to be significantly less than that in SPF mice in the duodenum and ileum, although not in the colon (25). These findings are consistent with our observations. Alternative or compensatory mechanisms must therefore exist for maintaining the integrity of the ENS when enteric bacteria and TLR4 signaling are permanently absent, which do not come into play when bacterial and TLR4 signaling are lost acutely.

In the intestinal tract, TLR4 has been reported to be expressed by enteric neurons (11), primary cultured enteric glia (12), and various immune cells (9) including dendritic cells. In the current study, we found most mucosal glia to be TLR4-immunoreactive in the colonic lamina propria.

The physiological effects of TLR4 signaling, particularly on enteric neurons and glia, remain incompletely understood. Using a culture system of enteric neurons isolated from the intestine, Anitha and colleagues showed that activation of TLR4 signaling with low-doses of LPS enhanced the viability of cultured enteric neurons via activation of NF-κB (13). Kovler and colleagues reported that mice lacking TLR4 on enteric glia retain their enteric glia but are protected from experimental necrotizing enterocolitis and do not develop dysmotility when experimental necrotizing enterocolitis is induced (26). Few effects of TLR4 signaling on mucosal glia, however, have previously been reported.

Previous reports have shown that LPS can be detected in plasma and that plasma levels of LPS are nearly identical in mice in both normal and inflammatory states (27); however, plasma LPS levels are dramatically reduced in ABX mice (13). It is thus possible that even under normal conditions, LPS crosses the intestinal epithelial barrier to gain direct access to enteric nerve process, mucosal glia, and immune cells and TLR4 in the lamina propria. Such translocated LPS may contribute to the maintenance or the outgrowth of enteric nerves by activating TLR4 that cells of the wall of the bowel express.

Due to their anatomical proximity in the colonic mucosa, some interaction between dendritic cells and enteric mucosal glia cannot be excluded. Therefore, it is speculated that in addition to the direct activation of mucosal glia by LPS, there may be an indirect pathway that activates mucosal glia via the TLR4 expressed by dendritic cells in the colonic mucosa. Enteric glia, furthermore, express major histocompatibility class II protein and various cytokines (3); therefore, mucosal glia have been postulated to interact with the enteric mucosal immune system. Taken together, these findings are compatible with the idea that mucosal glia respond to gut microbiota and bacterial products and signal to various immune cells in the mucosal immune system through dendritic cells; however, the mechanism by which LPS or bacteria in the intestinal lumen cross the intestinal epithelial cell layer, a robust barrier against foreign threats, to gain access to cells in the lamina propria is still unknown.

### 4.3 Neurotrophic factor CNTF in the gut

Neurotrophic factors regulate the development, survival, and maintenance of neurons, including enteric neurons (16); the families of such factors include the neurotrophins, which consists of NT-3, NGF, BDNF and NT-4/5; the neurotrophic cytokine family, including CNTF, IL-6, and LIF; and the GDNF family. With respect to the ENS, cultured enteric glia have been reported to express higher levels of mRNA encoding CNTF than the neurotrophic factors GDNF, NGF, NT-3, NT-4/5, LIF, and BDNF (17).

CNTF was discovered as a neurotrophic cytokine that exerts a potent survival effect on ciliary nerves in chickens (28). CNTF is now known to be a neurotrophic factor expressed in glial cells, astrocytes, Schwann cells, skeletal muscle, etc. (29). It has also been reported that in the CNS, CNTF expressed on glial cells facilitates neuronal survival and differentiation. In the intestine, CNTF has been reported to induce nuclear translocation of STAT3 in neurons, to increase numbers of identified neurons in a concentration-dependent manner, and to promote the development and differentiation of enteric neurons from cultured neural crest-derived progenitor cells (30). CNTF, furthermore, has been reported to be a more potent neurotrophic factor than NT-3 and additive with NT-3 in the same cultured neural progenitor cells (16). In the adult intestine, however, the expression of CNTF and the effects of CNTF on enteric neurons remain poorly understood.

In the present study, nerve fiber density and CNTF expression in the lamina propria of the proximal colon were decreased 6 hours after ABX administration, a time-course that is consistent with the suggestion that CNTF is involved in the maintenance of nerve fiber density in the proximal colon. The majority of CNTF immunoreactivity in the proximal colon was found to be present in mucosal glia and not in dendritic cells. ABX-induced dysbiosis, furthermore, resulted in a decrease in mucosal glia but not in dendritic cells. We therefore propose that dendritic cells, which are thought to be the primary cells for capturing luminal information in the intestine, are nevertheless not the cells that release CNTF to affect enteric nerve maintenance and outgrowth in the mucosa.

Kabouridis and colleagues have reported that ABX treatment for 3 weeks reduces the number of S100β-positive enteric glia within the mucosa of the small intestine of adult mice (8). In the small intestine of adult mice, furthermore, mucosal glia have been shown to be continuously renewed in a gut microbiota-dependent manner by incoming glial cells originating from within the enteric plexuses (8). These findings are consistent with the results of the present study showing a decrease in mucosal glia in ABX-induced dysbiosis after only 6 hours. Taken together, therefore, these findings suggest that new mucosal glia constantly migrate from the enteric plexuses to the mucosa in a gut microbiota-dependent manner and that these glial are needed to maintain the integrity of enteric neural circuits within the mucosa.

### 4.4 Crosstalk between enteric glia and neurons

The present study found that mucosal glia are morphologically located close to enteric nerve processes in the lamina propria of the murine proximal colon, which is consistent with previous findings (31). In addition, it has been reported that enteric glia promote the formation and functional maturation of enteric neural circuits in a coculture system of enteric glia and neurons derived from neural crest cells isolated from the intestines of fetal rats (32). *In vivo*, inhibition of enteric glial activation in mice has been shown to result in impaired motor function in the colon (33), suggesting that the failure of input from glia to enteric neurons may be involved in intestinal motility disturbances. It has also been reported that enteric glia are decreased in the myenteric plexus in patients with obstructive defecation, although the number of neurons in the myenteric plexus remain unchanged in that condition (34). Taken together, these observations suggest that enteric glia facilitate the formation and subsequent functional maturation of enteric neural circuits, which in turn regulate intestinal function.

We found synaptophysin1 immunoreactivity apparently located in mucosal glia, although the microscopic resolution was insufficient to distinguish between immunoreactivity within glial cytoplasm from that within nerve processes that glia envelop. As previously reported (21), synaptophysin1 immunoreactivities were also observed in neuroendocrine-like cells of the enteric epithelial cell layer, which indicates that synaptophysin1 immunohistochemistry can detect synapse-like structures in nonneuronal cells. Experiments were not designed to determine how enteric glia secrete peptides or products like CNTF; nevertheless, it is clear that nerve processes and glia interact and may do so through by a process that is similar to vesicular exocytosis, like synaptic transmission.

### 4.5 Involvement of CNTF released from mucosal glia in the formation and maintenance of mucosal nerve processes

The current study shows, for the first time in an *in vivo* model, that the ABX-induced reduction in nerve fiber density in the mucosa of the proximal colon is opposed and reversed to near-normal density by the co-administration of CNTF. We also showed for the first time that CNTF acts locally within the colon to increase nerve fiber density in an *in vitro* model using the isolated proximal colon of ABX mice.

It has previously been reported in an *in vitro* system using cultured dorsal root ganglion cells that CNTF partially restores neurotoxin-induced neurite retraction and exhibits neuroprotective effects (35); moreover, the neurites of cultured retinal ganglion cells have been reported to elongate upon treatment with CNTF (36). It has also been reported that, in an *in vivo* system, subconjunctival administration of antibodies to CNTF delays the regeneration of sensory neurons after injury to the cornea; conversely, CNTF administration promotes the regeneration of these sensory neurons and CNTF restores the reduced nerve density in the cornea due to diabetic sensory neuropathy to a level comparable to normal (37).

Based on previous reports and the effects found in the current investigation that CNTF promotes enteric nerve outgrowth in the colonic mucosa, CNTF can be considered to be an important neurotrophic factor that acts directly on enteric neurons. These results suggest that CNTF from mucosal glia is directly involved in the elongation of enteric nerve fibers, maintenance, repair, and function of mucosal neural circuits. In the lamina propria of the colon, therefore, mucosal glia may function to maintain the homeostasis of mucosal nerves in a process that depends upon gut microbiota, TLR4 signaling, and CNTF.

## 5 Conclusion

Whereas the CNS is covered and protected by the skull, spinal vertebrae, and cerebrospinal fluid, the ENS and its nerve processes are relatively exposed within gut. The lamina propria, through which many enteric nerve fibers ramify, is located at the interface of the body and the outside world, separated by only a single layer of epithelial cells. Throughout life, therefore, the ENS would be in constant danger from damage that translocation of luminal contents, including toxins, substances that produce inflammation, as well as pathogenic and commensal microorganisms, might cause. To appropriately respond to these threats, homeostatic mechanisms, such as strong enteric neural circuit repair mechanisms, are likely to have evolved. Although neuronal apoptosis occurs consistently in the adult normal intestine, recent studies have suggested a mechanism that maintains the homeostasis of the ENS through constitutive neurogenesis that complements enteric neuronal cell death (31). Little is known, however, about the mechanisms that maintain enteric nerve fibers and circuits in the ENS or within the lamina propria.

In the current study, we tested and provided evidence to support the hypothesis that under normal conditions, the constitutive activation of TLR4 signaling by enteric bacteria maintains the localization of mucosal glia in the lamina propria and their expression of CNTF; moreover, glial-derived CNTF, secreted from mucosal glia in response to TLR4 signaling maintains the integrity of enteric nerve fibers within the colonic mucosa.

The observation that the symbiotic relationship between components of the ENS and foreign gut microbiota maintains homeostasis of mucosal nerves, an indispensable process for life, is quite surprising. Indeed, it has been reported that the composition of fecal microbiota is different in pediatric patients with constipation from that in healthy control children (38), suggesting the potential for interactions between gut microbiota and the ENS have the potential to alter gut function.

In the future, it will be necessary to find answers to questions such as how (in addition to TLR4 signaling) mucosal glia recognize enteric bacteria, how CNTF released from mucosal glia assembles and maintains enteric processes, how (besides signaling with CNTF) mucosal glia communicate with enteric neurons, and what the physiological and pathophysiological roles of mucosal glia play in the intestine. Answers to these questions may facilitate further understanding of the etiology, pathology, and possible novel treatment of functional and inflammatory intestinal diseases, such as IBS and IBD. Recently, it has been reported that the subepithelial enteric glial cell network in close proximity to the damaged epithelium plays a critical role in promoting mucosal barrier restoration as a key regulator of the mucosal barrier (39). This observation supports the idea that mucosal glia play an important role in preserving and maintaining the homeostasis of the bowel.

## Data Availability

The original contributions presented in the study are included in the article/**Supplementary Material**. Further inquiries can be directed to the corresponding author.

## Supplementary material

Supplementary Figure 1 Quantification and normalization of tubulin-β3-immunoreactive enteric nerve fibers. Images taken by a confocal microscope with the same settings were loaded into ImageJ and analyzed by binarizing the images with the same thresholding conditions. Mucosal areas were manually selected in these images and analyzed for the positive percentage of tubulin-β3 in the mucosal area. For illustrative purposes, we used **(B)** and **(C)** in **Figure 1**. The photographs shown below each of **Figures 1B** and **C** are the photographs used for analysis. The photographs were binarized, with tubulin-β3 positivity indicated in red and tubulin-β3 negativity indicated in black. The area surrounded by white dots and yellow lines is the mucosa. The percentage of positive (red) area within the mucosal area was calculated.

Supplementary Figure 2 Effects of ABX-induced dysbiosis on cecal weight. To investigate the effect of gut microbiota on enteric mucosal nerve fibers, ABX were orally administered once daily to SPF mice to induce dysbiosis (ABX mice). Cecal weights **(A–E)** at each test time point are shown; starting at 6 hours, enlargement of the cecum was observed in ABX mice, and the cecum was significantly heavier in ABX mice than in vehicle-treated mice. ***P* < 0.01 vs. vehicle mice. *N* = 10–12.

Supplementary Figure 3 Verification of the effect of oral administration of ABX (6 hours) on enteric nerve fibers in the mucosal lamina propria of the proximal colon of mice by immunohistochemistry using an antibody against the neural marker PGP9.5. Sections of the proximal colon of vehicle-treated and ABX mice were immunostained with antibodies to the neural marker PGP9.5. Typical images of PGP9.5-immunoreactive nerves at 6 hours after ABX administration are shown (**A**: vehicle mice; **B**: ABX mice). Bars show the nerve fiber density (% area) in the lamina propria. The nerve fiber density was significantly lower in ABX-treated mice than in vehicle-treated mice. Scale bar = 50 µm, ***P* < 0.01 vs. vehicle mice. *N* = 6–10.

Supplementary Figure 4 Enteric nerve fibers in the lamina propria of the proximal colon three days after a single dose of ABX. Sections of the proximal colon of vehicle-treated and ABX mice were immunostained with antibodies to tubulin-β3. Images were acquired using a Zeiss LSM780 laser-scanning confocal microscope. Typical image of tubulin-β3-immunoreactive nerve fiber recovery is shown in ABX mice **(B)**: the density of enteric nerve fibers, which was reduced after a single dose of ABX, recovered on day 3 to almost the same level as that in vehicle-treated normal mice **(A)**. Scale bar = 50 µm.

Supplementary Figure 5 Effects of the gut microbiota on body weight, cecal weight and colon length in germ-free mice. To investigate the effect of the gut microbiota on enteric mucosal nerve fibers, we used germ-free mice (GF mice). GF mice were slightly heavier than SPF mice (**A**; **P* < 0.05; *n* = 6). The cecal weight in GF mice was approximately 25 times greater than that of SPF mice (**B**; ***P* < 0.01; *n* = 6). The length of the colon of GF mice was also greater than that of SPF mice (**C**; ***P* < 0.01; *n* = 5–6).

Supplementary Figure 6 Effects of TLR4 inhibitor on cecal weight. We investigated the effect of TLR4 signaling on enteric mucosal nerve fibers. To do so, we administered TAK242, a TLR4 inhibitor, to SPF mice. Twenty-four hours after administration, the cecal weight of TAK242-treated mice was not significantly difference from that of vehicle-treated mice. *N* = 13–14.

Supplementary Figure 7 Effect of TLR4 deficiency on body weight, cecal weight and colon length. We investigated enteric mucosal nerve fibers in TLR4-deficient C57BL/6 mice (*Tlr4*-/- mice) and SPF C57BL/6 mice (WT mice). *Tlr4*-/- mice were slightly heavier than SPF mice (**A**; ***P* < 0.01). The cecal weight of *Tlr4*-/- mice tended to be greater than that of WT mice, but the difference was not significant **(B)**. No significant difference in colon length was observed between WT mice and *Tlr4*-/- mice **(C)**. *N* = 4.

Supplementary Figure 8 Expression of CNTF immunoreactivity in mucosal glia of the lamina propria of the mouse proximal colon. Sections of the proximal colon of SPF mice were doubly immunostained with antibodies to CNTF and CD11c. Images were acquired using a Zeiss LSM700 laser-scanning confocal microscope. Typical merged images with antibodies to CNTF and antibodies to CD11c in the lamina propria are shown. No expression of CNTF (red) immunoreactivity was observed in CD11c (green)-immunoreactive dendritic cells **(C)**. Scale bar = 10 µm.

Supplementary Figure 9 Anatomical proximity between mucosal glia, enteric nerve fibers and CD11c-immunoreactive dendritic cells in the lamina propria of the proximal colon of SPF mice. Sections of the proximal colon of SPF mice were doubly immunostained with antibodies to S100β and synaptophysin1 or with antibodies to S100β and CD11c. Images were acquired using a Zeiss LSM700 laser-scanning confocal microscope. Typical merged images with S100β (red) and synaptophysin1 (green) immunoreactivities are shown in **(A, B)**, and those with S100β (red) and CD11c (green) immunoreactivities are shown in **(C, D)**. Arrows (in **A, B**) indicate neuroendocrine-like cells of the enteric epithelial cell layer. Scale bar = 50 µm.

Supplementary Figure 10 Effects of ABX-induced dysbiosis on dendritic cells in the lamina propria of the mouse proximal colon. Sections of the proximal colon of vehicle-treated and ABX mice were immunostained with antibodies against CD11c and F4/80. Images were acquired using a Zeiss LSM780 laser-scanning confocal microscope. Typical images of CD11c immunoreactivities are shown in vehicle-treated mice (aa) and ABX mice (ba). Typical images of F4/80 immunoreactivities are shown in vehicle-treated mice (ab) and ABX mice (bb). Double immunostaining revealed that there is no difference in the number of CD11c-positive-F4/80-negative dendritic cells between vehicle-treated mice (ac) and ABX mice (bc). Bars indicate the number of CD11c-positive-F4/80-negative dendritic cells **(C)**. Scale bar = 50 µm. *N* = 6.

Supplementary Figure 11 Effect of co-administration of CNTF and ABX on cecal weight. To investigate the effect of CNTF on enteric mucosal nerve fibers in ABX mice, CNTF was administered subcutaneously with simultaneous oral administration of ABX (ABX+CNTF mice). The cecal weight of ABX and ABX+CNTF mice was significantly greater than that of vehicle-treated mice 6 hours later, but no other morphological effect of CNTF administration on the cecum was observed. ***P* < 0.01 between vehicle mice and ABX mice, and between vehicle mice and ABX+CNTF mice. *N* = 6–7.
