# Supplementary figures and images for: Indigenous gut microbiota constitutively drive release of ciliary neurotrophic factor from mucosal enteric glia to maintain the homeostasis of enteric neural circuits
Source: Front Immunol. 2024 Nov 13;15:1372670. doi: 10.3389/fimmu.2024.1372670 (PMC11598343; doi:10.3389/fimmu.2024.1372670)

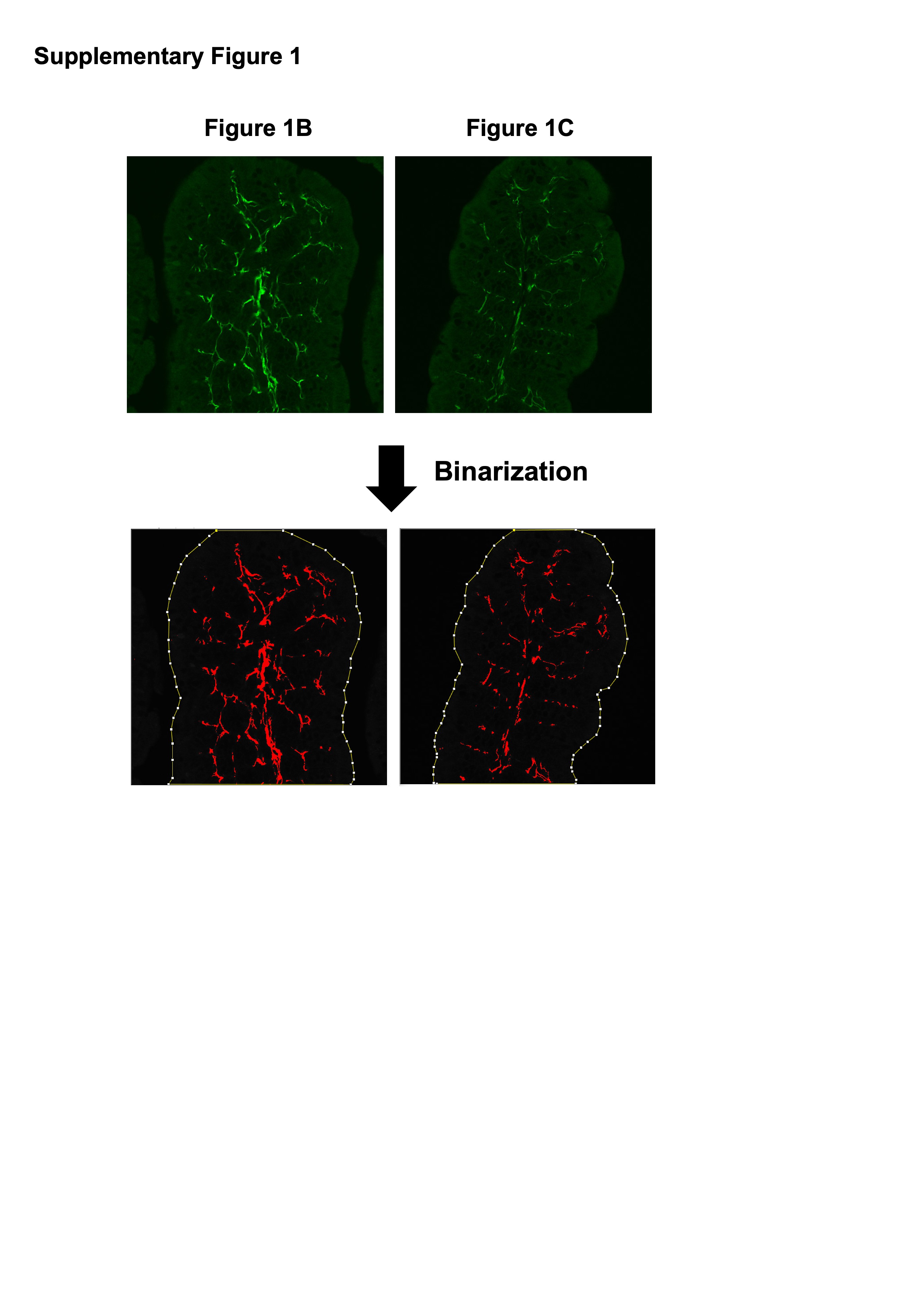

Supplement: Supplementary Figure 1 — Quantification and normalization of tubulin-β3-immunoreactive enteric nerve fibers. Images taken by a confocal microscope with the same settings were loaded into ImageJ and analyzed by binarizing the images with the same thresholding conditions. Mucosal areas were manually selected in these images and analyzed for the positive percentage of tubulin-β3 in the mucosal area. For illustrative purposes, we used (B) and (C) in Figure 1 . The photographs shown below each of Figures 1B and C are the photographs used for analysis. The photographs were binarized, with tubulin-β3 positivity indicated in red and tubulin-β3 negativity indicated in black. The area surrounded by white dots and yellow lines is the mucosa. The percentage of positive (red) area within the mucosal area was calculated. [file Image1.jpeg]

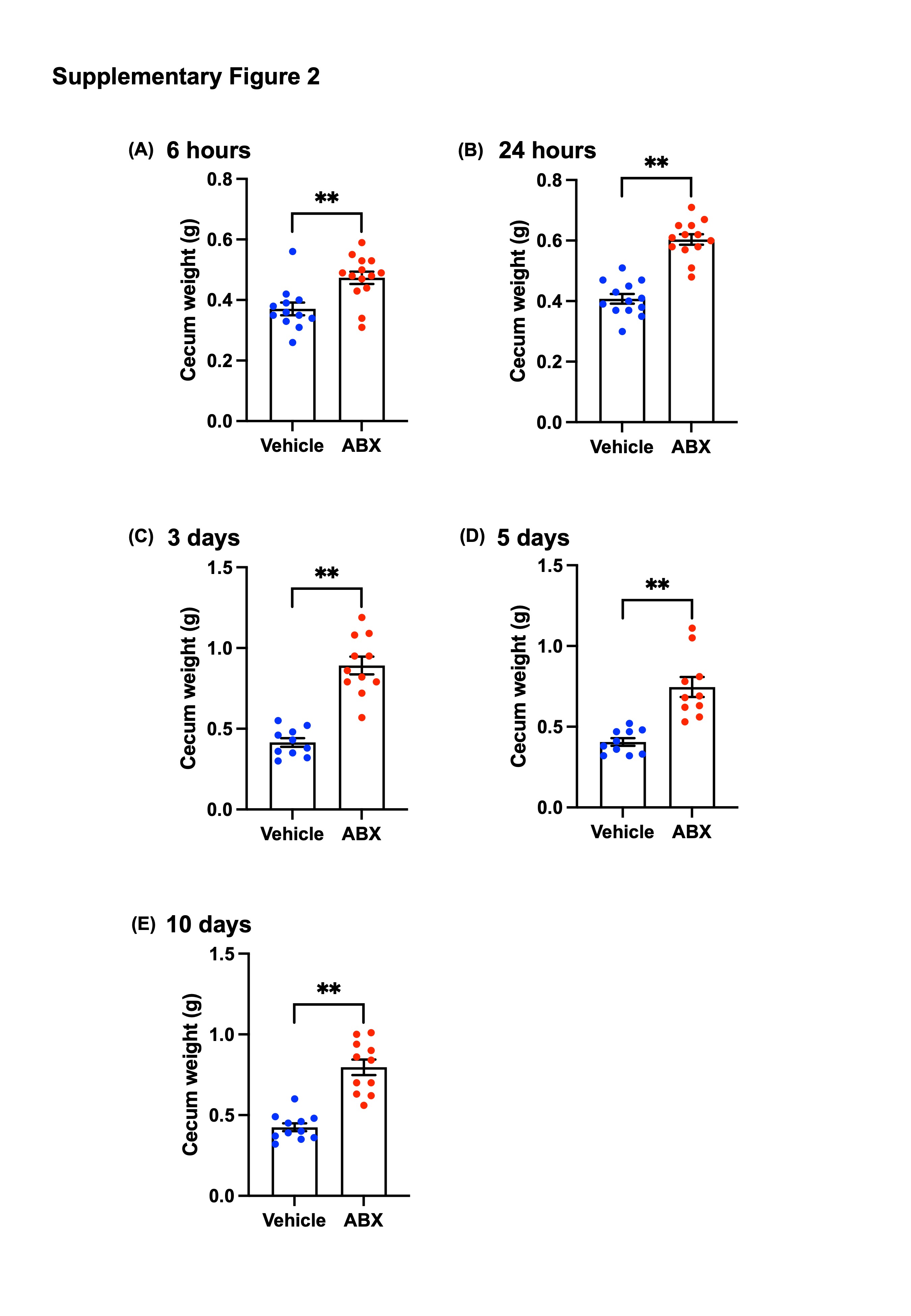

Supplement: Supplementary Figure 2 — Effects of ABX-induced dysbiosis on cecal weight. To investigate the effect of gut microbiota on enteric mucosal nerve fibers, ABX were orally administered once daily to SPF mice to induce dysbiosis (ABX mice). Cecal weights (A–E) at each test time point are shown; starting at 6 hours, enlargement of the cecum was observed in ABX mice, and the cecum was significantly heavier in ABX mice than in vehicle-treated mice. **P < 0.01 vs. vehicle mice. N = 10–12. [file Image2.jpeg]

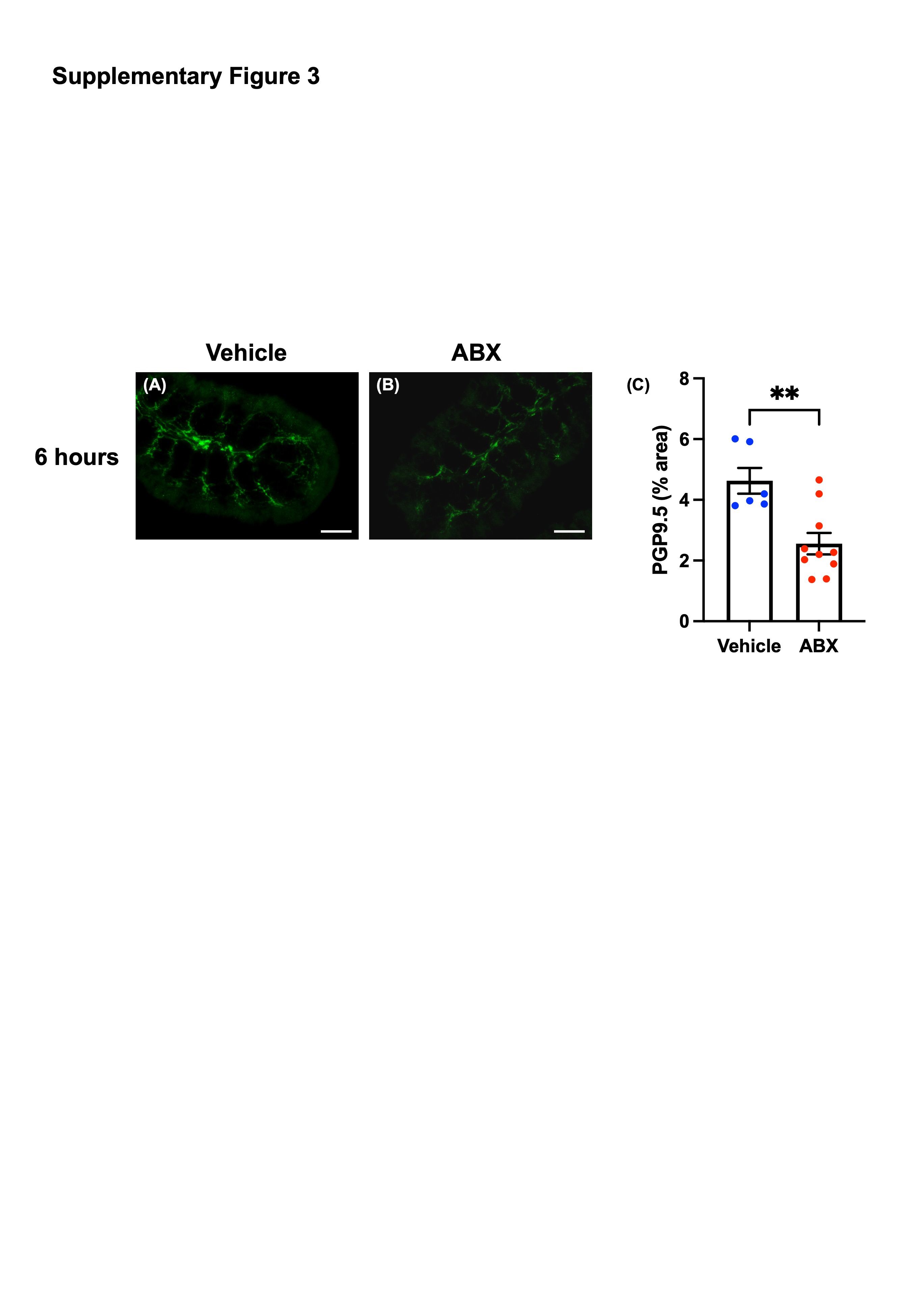

Supplement: Supplementary Figure 3 — Verification of the effect of oral administration of ABX (6 hours) on enteric nerve fibers in the mucosal lamina propria of the proximal colon of mice by immunohistochemistry using an antibody against the neural marker PGP9.5. Sections of the proximal colon of vehicle-treated and ABX mice were immunostained with antibodies to the neural marker PGP9.5. Typical images of PGP9.5-immunoreactive nerves at 6 hours after ABX administration are shown (A: vehicle mice; B: ABX mice). Bars show the nerve fiber density (% area) in the lamina propria. The nerve fiber density was significantly lower in ABX-treated mice than in vehicle-treated mice. Scale bar = 50 µm, **P < 0.01 vs. vehicle mice. N = 6–10. [file Image3.jpeg]

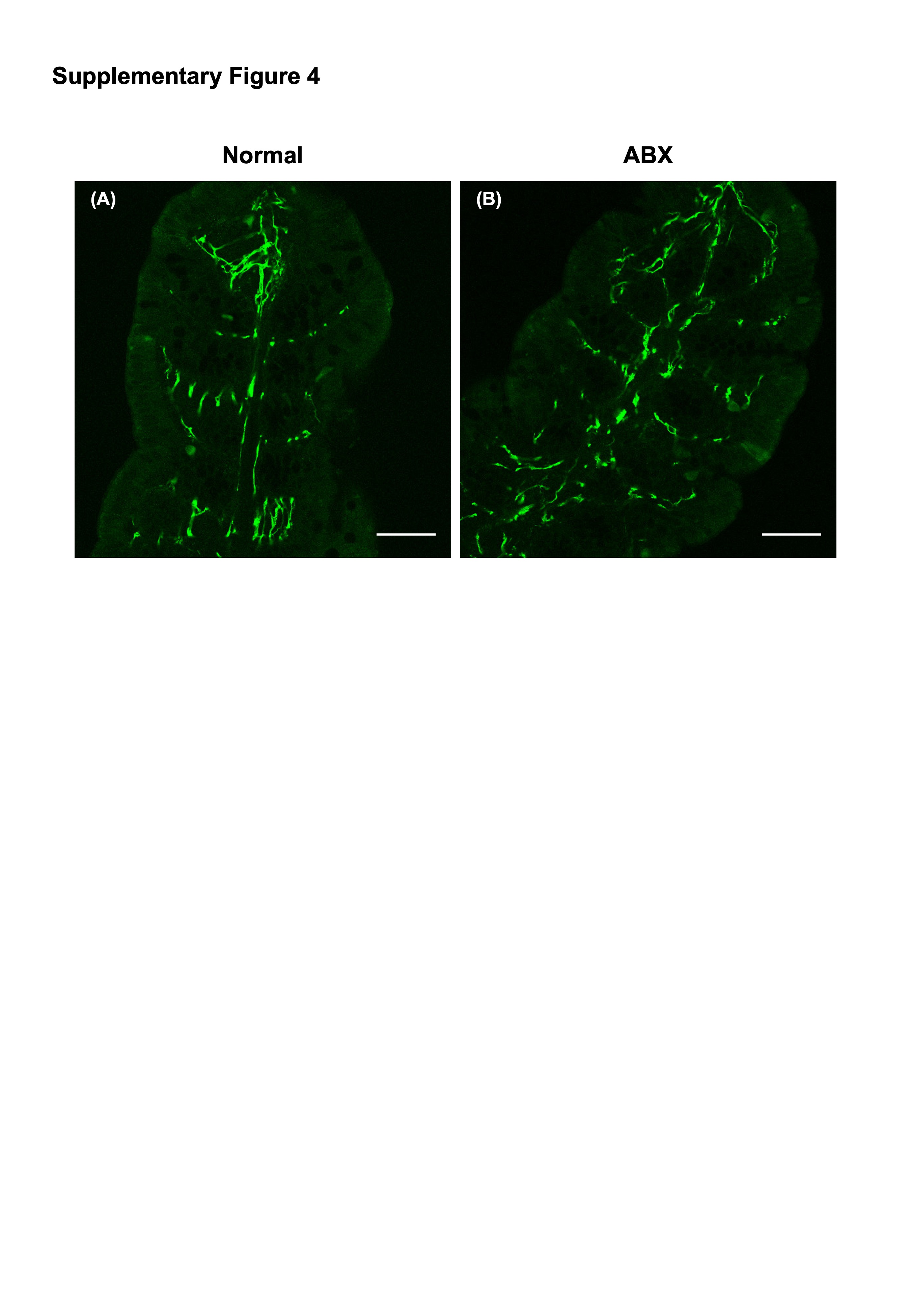

Supplement: Supplementary Figure 4 — Enteric nerve fibers in the lamina propria of the proximal colon three days after a single dose of ABX. Sections of the proximal colon of vehicle-treated and ABX mice were immunostained with antibodies to tubulin-β3. Images were acquired using a Zeiss LSM780 laser-scanning confocal microscope. Typical image of tubulin-β3-immunoreactive nerve fiber recovery is shown in ABX mice (B): the density of enteric nerve fibers, which was reduced after a single dose of ABX, recovered on day 3 to almost the same level as that in vehicle-treated normal mice (A). Scale bar = 50 µm. [file Image4.jpeg]

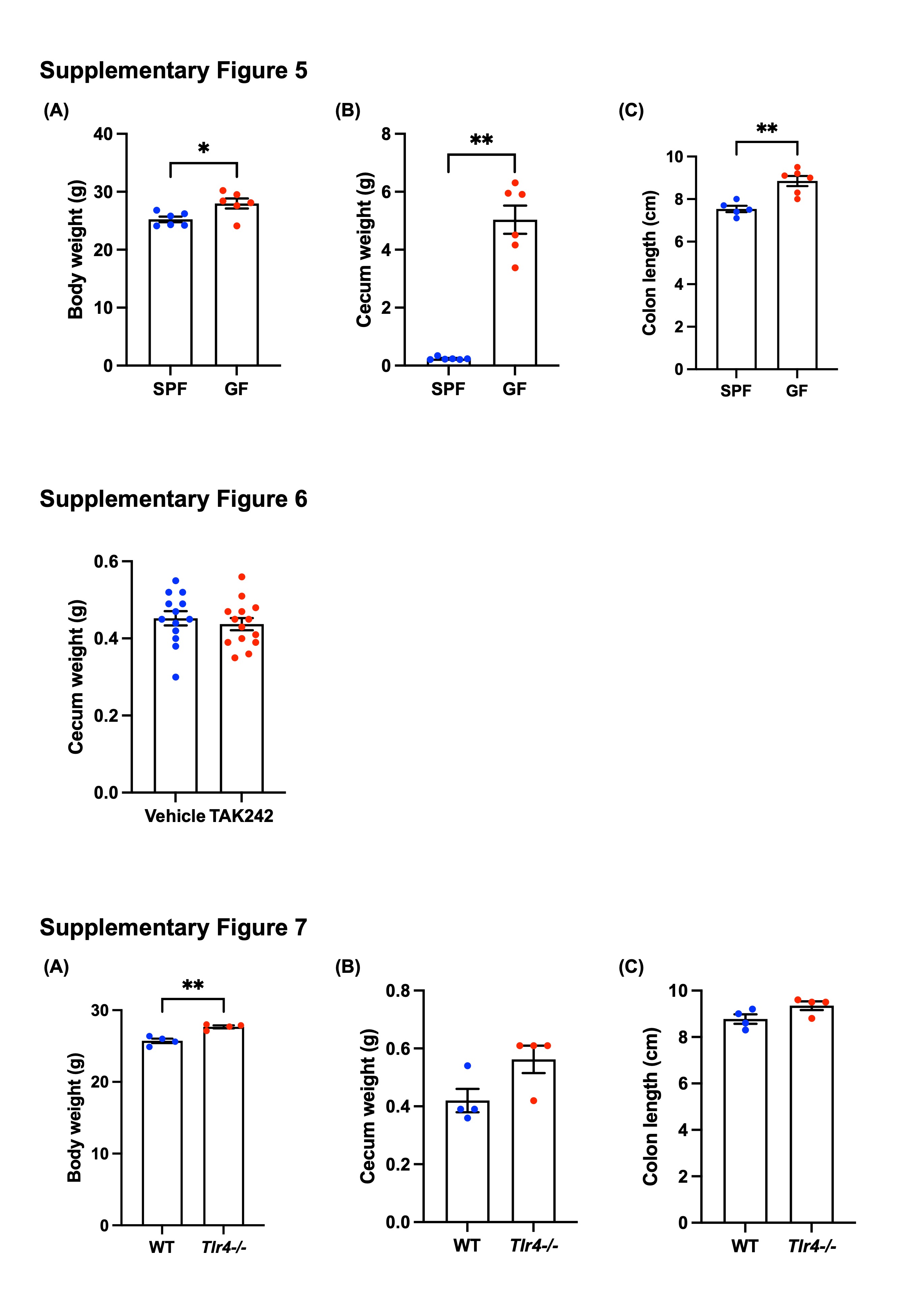

Supplement: Supplementary Figure 5 — Effects of the gut microbiota on body weight, cecal weight and colon length in germ-free mice. To investigate the effect of the gut microbiota on enteric mucosal nerve fibers, we used germ-free mice (GF mice). GF mice were slightly heavier than SPF mice (A; *P < 0.05; n = 6). The cecal weight in GF mice was approximately 25 times greater than that of SPF mice (B; **P < 0.01; n = 6). The length of the colon of GF mice was also greater than that of SPF mice (C; **P < 0.01; n = 5–6). [file Image5.jpeg]

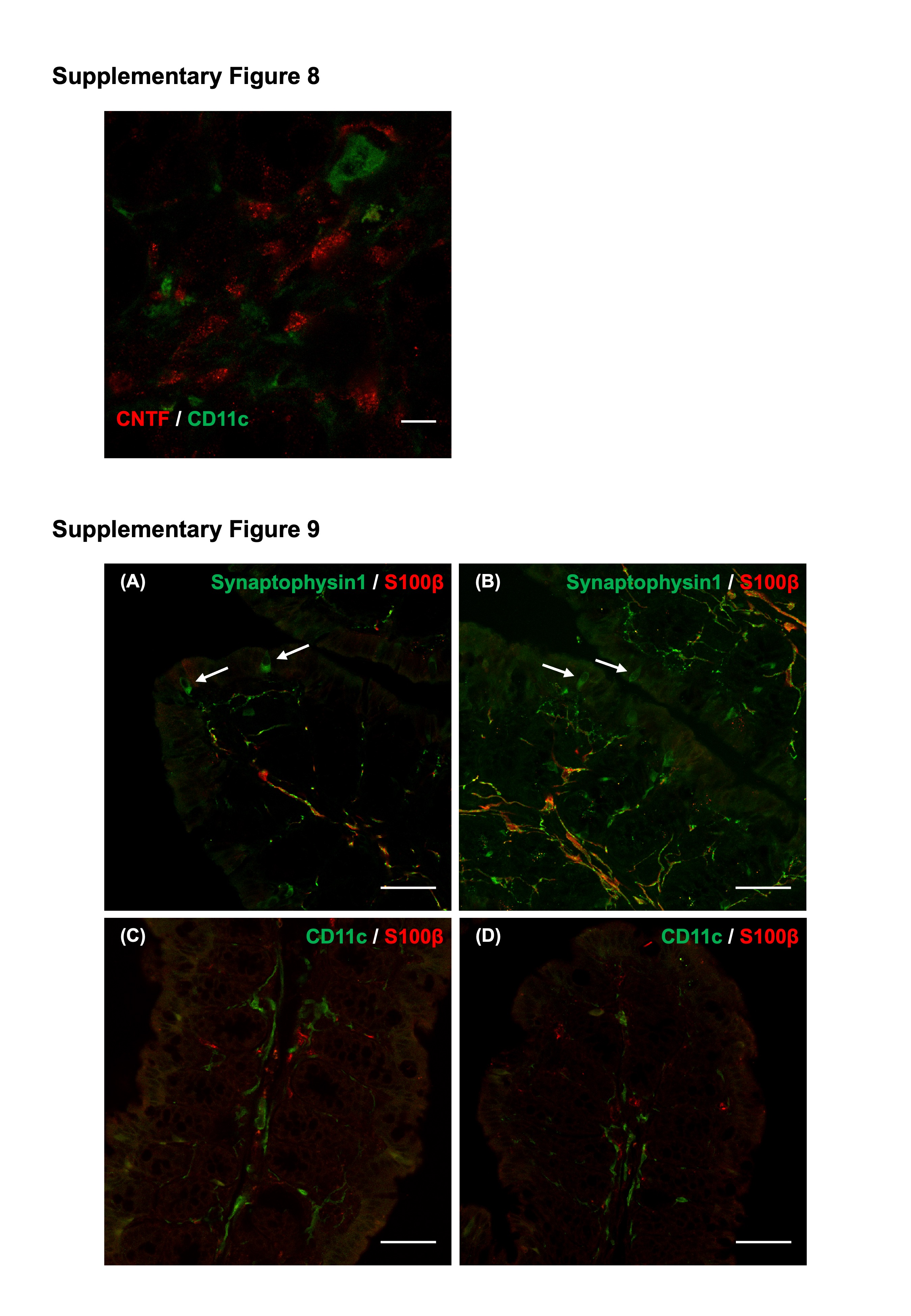

Supplement: Supplementary Figure 8 — Expression of CNTF immunoreactivity in mucosal glia of the lamina propria of the mouse proximal colon. Sections of the proximal colon of SPF mice were doubly immunostained with antibodies to CNTF and CD11c. Images were acquired using a Zeiss LSM700 laser-scanning confocal microscope. Typical merged images with antibodies to CNTF and antibodies to CD11c in the lamina propria are shown. No expression of CNTF (red) immunoreactivity was observed in CD11c (green)-immunoreactive dendritic cells (C). Scale bar = 10 µm. [file Image6.jpeg]

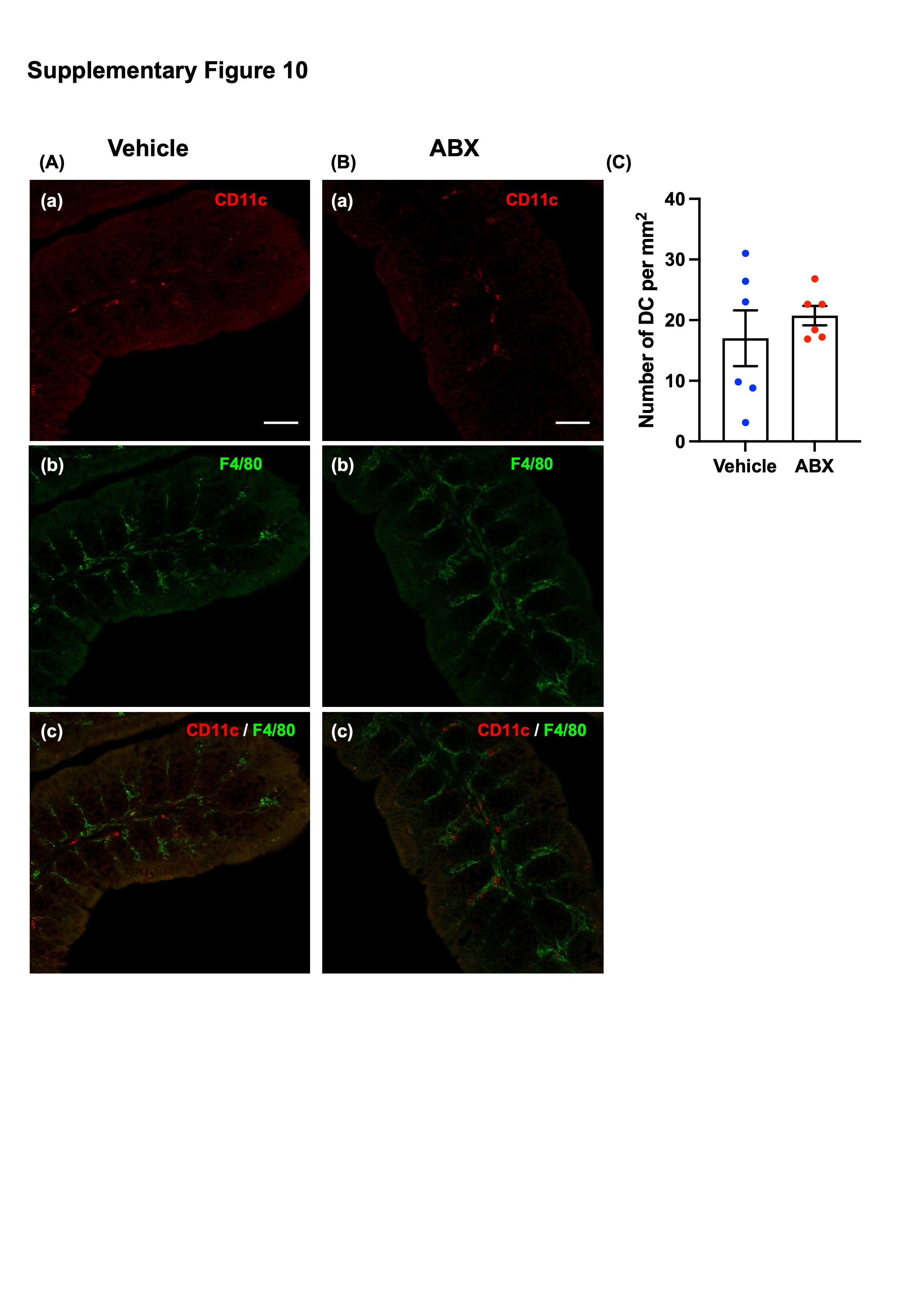

Supplement: Supplementary Figure 10 — Effects of ABX-induced dysbiosis on dendritic cells in the lamina propria of the mouse proximal colon. Sections of the proximal colon of vehicle-treated and ABX mice were immunostained with antibodies against CD11c and F4/80. Images were acquired using a Zeiss LSM780 laser-scanning confocal microscope. Typical images of CD11c immunoreactivities are shown in vehicle-treated mice (aa) and ABX mice (ba). Typical images of F4/80 immunoreactivities are shown in vehicle-treated mice (ab) and ABX mice (bb). Double immunostaining revealed that there is no difference in the number of CD11c-positive-F4/80-negative dendritic cells between vehicle-treated mice (ac) and ABX mice (bc). Bars indicate the number of CD11c-positive-F4/80-negative dendritic cells (C). Scale bar = 50 µm. N = 6. [file Image7.jpeg]

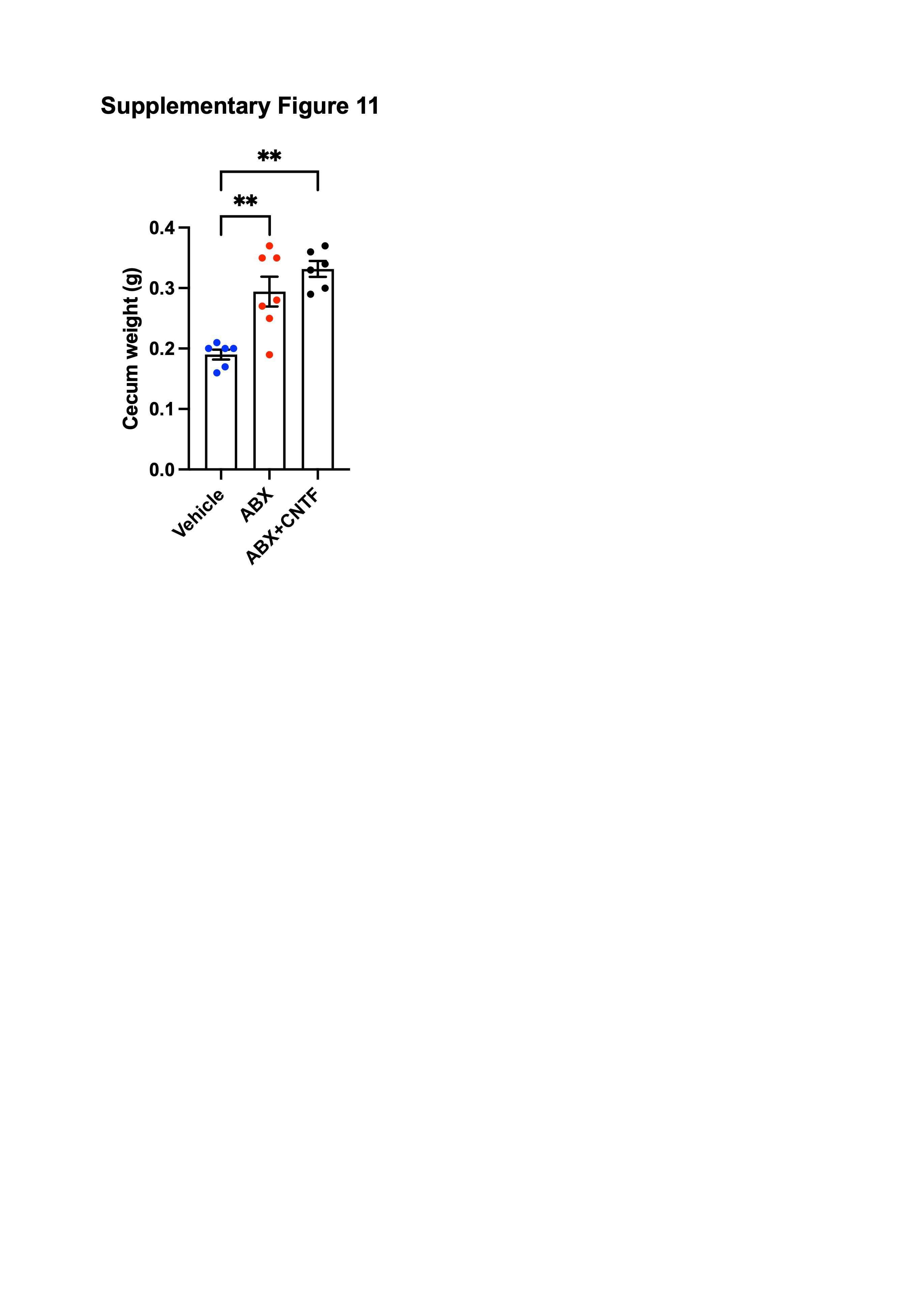

Supplement: Supplementary Figure 11 — Effect of co-administration of CNTF and ABX on cecal weight. To investigate the effect of CNTF on enteric mucosal nerve fibers in ABX mice, CNTF was administered subcutaneously with simultaneous oral administration of ABX (ABX+CNTF mice). The cecal weight of ABX and ABX+CNTF mice was significantly greater than that of vehicle-treated mice 6 hours later, but no other morphological effect of CNTF administration on the cecum was observed. **P < 0.01 between vehicle mice and ABX mice, and between vehicle mice and ABX+CNTF mice. N = 6–7. [file Image8.jpeg]
